# Design and implementation of an adaptive fuzzy sliding mode controller for drug delivery in treatment of vascular cancer tumours and its optimisation using genetic algorithm tool

**DOI:** 10.1049/syb2.12051

**Published:** 2022-09-30

**Authors:** Ehsan Sadeghi Ghasemabad, Iman Zamani, Hami Tourajizadeh, Mahdi Mirhadi, Zahra Goorkani Zarandi

**Affiliations:** ^1^ Electrical and Electronic Engineering Department Shahed University Tehran Iran; ^2^ Department of Mechanical Engineering Faculty of Engineering Kharazmi University Tehran Iran; ^3^ Electrical and Electronic Engineering Department Kharazmi University Tehran Iran; ^4^ Department of Mechanical Engineering Yazd University Yazd Iran

**Keywords:** anti‐angiogenic, cancer tumour, chemotherapy, fuzzy tuner, genetic optimisation tool, input–output feedback linearisation, sliding mode control, adaptive control, cancer, closed loop systems, drug delivery systems, fuzzy control, genetic algorithms, patient treatment, radiation therapy, robust control, tumours, variable structure systems

## Abstract

In this paper, the side effects of drug therapy in the process of cancer treatment are reduced by designing two optimal non‐linear controllers. The related gains of the designed controllers are optimised using genetic algorithm and simultaneously are adapted by employing the Fuzzy scheduling method. The cancer dynamic model is extracted with five differential equations, including normal cells, endothelial cells, cancer cells, and the amount of two chemotherapy and anti‐angiogenic drugs left in the body as the engaged state variables, while double drug injection is considered as the corresponding controlling signals of the mentioned state space. This treatment aims to reduce the tumour cells by providing a timely schedule for drug dosage. In chemotherapy, not only the cancer cells are killed but also other healthy cells will be destroyed, so the rate of drug injection is highly significant. It is shown that the simultaneous application of chemotherapy and anti‐angiogenic therapy is more efficient than single chemotherapy. Two different non‐linear controllers are employed and their performances are compared. Simulation results and comparison studies show that not only adding the anti‐angiogenic reduce the side effects of chemotherapy but also the proposed robust controller of sliding mode provides a faster and stronger treatment in the presence of patient parametric uncertainties in an optimal way. As a result of the proposed closed‐loop drug treatment, the tumour cells rapidly decrease to zero, while the normal cells remain healthy simultaneously. Also, the injection rate of the chemotherapy drug is very low after a short time and converges to zero.

## 1 INTRODUCTION

Cancer is the second leading cause of death in the world [1]. Since 2012, there have been 8.2 million deaths per year due to cancer. There are several common treatments for cancer, including surgery, radiation therapy, immunotherapy, and chemotherapy, each of which has a lot of significant side effects. Chemotherapy is one of the most important and effective ways to treat cancer. Since the dose of the drug and the response of the patient to this treatment is not explicitly determined and similar for everybody, the efficiency of the treatment and its related side effects are not unique for everybody and cannot be deterministically modelled. Extracting a mathematical model of the body response to the chemotherapy treatment can significantly increase its efficiency and reduce its relevant side effects. Different mathematical models have been proposed so far for the treatment of cancer with the aid of chemotherapy in recent years. However, the fundamental challenge is to extract a precise model of the dynamics of the healthy and cancerous cells. The main challenges of defining a general model and designing an efficient controller are, the side effects of chemotherapy, its long duration of treatment, parametric uncertainty of each body with respect to this controlling drug, and affectability of the body from environmental disturbance and situations. As a result, the target of this research is to design an appropriate and functional control that can cure the disease in the shortest time with the least amount of injection and side effects in a robust way.

The first efficient step towards controlling the cancer growth in an analytic way is to model the behaviour of the cancer dynamic. For the first time, H Skipper has developed a mathematical model to calculate the rate of chemotherapy drug distribution based on the tumour volume [2]. Afterwards, other models including EMAX and Norton–Simon proposed by Kohandel et al., considered other factors such as the effect of obesity and the rate of chemotherapy drug injection, which are based on the tumour growth, rather than its volume [3, 4]. Other mathematical models are also presented so far, for which in addition to the system dynamic analysis, the biological concept of the equilibrium points together with their related optimal control signal are also obtained by defining a target function. Judah Folkman has proposed, for the first time, the hypothesis that tumour growth is dependent on angiogenesis [5]. Subsequent research has shown that the growth development of the tumour metastases can be intensified by the creation of new veins and providing the nutrient requirements of the tumour. This fact was firstly approved in 2004 for the treatment of colorectal cancer using anti‐angiogenesis. They were then developed either as a monotherapy drug or in combination with other cytotoxic and chemotherapy drugs. A. D'Onofrio et al. designed a mathematical model with four common differential equations and defined its corresponding anti‐angiogenic drug and chemotherapy as a cancer treatment. Here for the first time, the optimal doses of the chemotherapy were obtained to minimise the maximum tumour size for the last day of the treatment process [6]. A more comprehensive model for exploring the dynamics of the cancer cells was defined by STR Pinho et al. In this model, the effects of five variables, including natural, cancer, and endothelial cells, along with chemotherapy and anti‐angiogenesis drugs in the body, have been considered [7]. Mathematical analysis of the untreated model showed that if the system is left untreated, the system tends to increase the cancer cells and reduce the normal cells, leading to death. This fact suggests that the patient's immune system cannot cure the cancer individually, and thus it is vital to employ supplementary methods for treating the cancer. In the following sections, it is explained that if an anti‐angiogenic drug, which only affects the endothelial cells, is injected alone, it does not cure the patient. In some cases, single chemotherapy may cure the patient under certain conditions. The mentioned extracted mathematics models have shown that if both drugs are used simultaneously, the individual is more likely to be recovered.

In the above mentioned research, a general multivariable controlling strategy is not designed and implemented on the extracted analytic dynamic of cancer. Therefore, the efforts were then focussed on controlling the extracted dynamics of the cancer model. In Ref. [8], immunotherapy is employed to cure cancer using three states, including the activated immune‐system cells (commonly called effective cells), cancer cells, and the injection rate. The Input–Output feedback linearisation controller was designed then by Chien et al. for the same dynamics using the same therapy method. It should be noted that in these two articles, the patient will be fully recovered at infinite time and the cancer cells are not destroyed completely during a limited period of time [9]. However, Ref. [10] have designed an adaptive fuzzy back‐stepping control that destroys the cancer cells over a finite limited time. The above mentioned studies can decrease the cancer cells to zero over an infinite time while Ref. [11] does it during a limited time. Here the non‐linear portion of the system model is approximated by a fuzzy model. The effect of cytokine on cancer cell growth was modelled in Ref. [12]. In this model, just one type of immune system had been considered, that is Lanphosit B. In 2003, it was explored that the destruction of the cancer cells is proportional to the tumour growth rather than the tumour volume. Most of the mentioned controlling strategies are not robust, while environmental disturbances as a result of other drug sources or parametric uncertainties, which is due to imperfect modelling of the tumour, decrease the efficiency of the cancer controlling process. Sliding mode theory is one of the best candidates for robust control of such imperfect models. In 2015, Baghernia et al. presented a non‐linear predator‐predator model that shows the mutual effect between tumour and immune cells. The control model presented is based on a sliding state that is resistant to parametric uncertainty and converts the unstable states into desirable turbulent states [13]. In 2016, Hashemi et al. presented a comprehensive mathematical model for cancer treatment that investigates the parameters of chemistry, radiotherapy, and metastasis. In order to examine the model in the presence of the destructive effects of chemotherapy, the sliding controller is used to stabilise the system around the related equilibrium points [14]. In 2017, Shahri et al. Considered immunotherapy as an effective method for the treatment of cancer. Considering the uncertainties of the tumour growth model, a sliding controller is designed based on Lyapunov theory to achieve the appropriate dose in immunotherapy [15]. In 2018, Khalili examined the effect of obesity on tumour progression with optimal chemotherapy control. Here using the sliding surface, the optimal dosage of the drug to follow the desired path of the states is extracted [16]. Some other useful controlling strategies are presented so far, which are not yet employed for biological systems or cancer models. A control based on the passivity cascade technique is presented in Ref. [17] for the non‐linear inverse pendulum. The target is to stabilise the system in the presence of parametric uncertainties and disturbances. A novel sliding mode controller is proposed for the Bridge‐type Fault Current Limiter (BFCL) system in order to Fault Ride‐Through (FRT) of Doubly Fed Induction Generator (DFIG) in low voltage cases. It is shown here that BFCL based on Sliding Mode Control (SMC) has a better performance compared to BFCL, which is designed using an ordinary PI controller [18]. Another sliding mode controller is designed for wind turbines with variable speeds, based on Permanent Magnet Synchronous Generator (PMSG). The proposed controller is compared with simple PI and the first‐order sliding mode controller to show its superiority in DC voltage setting. It is also proved that this controller is more suitable for variable speed turbines [19]. In Ref. [20], a serial second‐order sliding mode controller based on Layer Management Interface (LMI) is proposed for controlling the non‐linear systems in the presence of parametric uncertainties and external disturbances. Here it is shown that the proposed method is not affected by chattering and is applicable to many Multi Input Multi Output (MIMO) systems.

The above‐mentioned studies cannot optimise the therapy efficiency, such as minimising the treatment period or its related side effects since no optimization tool is engaged. There are some studies in which an objective function is considered, and its value is minimised using a proposed optimal control. Martin et al. considered the final volume of the tumour as the related cost function and controlled cancer using the parametric control method and considered the side effects of the drug as the constraint of the system [21]. Afterwards, the Gompertez model was employed in [21] and the objective function was defined as a compromise between the drug injection and the tumour volume. P. Khalili et al. designed an optimal sliding mode controller in 2018, concerning the effects of obesity on cancer cells. In this article, chemotherapy injection together with anti‐angiogenic are used as the controlling inputs. In this paper, the Pinho model with five states is considered [22]. Fuzzy controller was used by the same authors for controlling the rate of the chemotherapy injection based on the Pinho model, while the amount of anti‐angiogenic was constant here, which means that no control signal is designed for this input [22]. Contrary to this study, Khalili et al. in Ref. [23] have extracted the optimal path and an adaptive controller for drug delivery to a cancerous tumour in chemotherapy was designed in which the anti‐angiogenic input is not constant and its dynamic is considered. Here the cost function is minimising the death of the normal cell during the chemotherapy using the steepest decent algorithm. Afterwards, this concept was redesigned and a non‐linear optimal adaptive controller was implemented for the mentioned model, which reduced the amount of the cancer cells to zero for more than 100 days [22]. Mital et al., have used a genetic algorithm to find the optimal solution for heat damage of cancer tissues. Radiotherapy is employed in this study and the optimal temperature of cancer treatment is investigated here using the genetic algorithm [24]. In 2020, Karar et al. proposed an Intuitionistic Fuzzy Logic Controller (IFLC) for the treatment of cancer in the presence of immune system limitations. An anti‐cancer drug delivery system is proposed here that is optimised and adapted based on the Invasive Weed Optimisation (IWO) algorithm [25]. Potts provided a real‐time fuzzy tuning system of industrial PID controllers and delivered an analysis of the treatment response of a PID controller equipped with a fuzzy automatic tuner [26]. Zhou used an organism search algorithm for controller tuning process and parameter controller adjustment and performed many experiments based on this system [27]. In 2020, Van designed a robust controller for a robot in order to provide uniformity between Self‐Tuning Fuzzy Proportional‐Integral‐Derivative (PID) Non‐singular Fast Terminal Sliding Mode Control (STF‐PID‐NFTSM) and Time Delay Estimation (TDE). The proposed method was compared with conventional PID‐NFTSMC as well as up‐to‐date FTC methods [28]. Finally, Ref. [29] is about experimental data related to chemotherapy reform by using anti‐angiogenic drugs. A fuzzy controller is designed in Ref. [30] in order to destroy the cancer cells in a finite time. In Ref. [31], it has been considered an optimal method for cancerous tumour by anti‐angiogenic and chemotherapy.

According to the above explanations, it can be seen that in cancer treatment, not only should cancer cells be considered but also the endothelial cells parameter should be considered to improve cancer and reduce the treatment time. In the above studies, the reduction of the side effects of chemotherapy using anti‐angiogenic is not maximised using an efficient optimisation tool in a robust way. To meet this goal, both the chemotherapy and anti‐angiogenic drugs are used here together for the system defined by the STR Pinho model in order to decrease the chemotherapy side effects. Therefore, it is proposed to employ two input signals to treat cancer including chemotherapy and anti‐angiogenic. Two non‐linear controllers of feedback linearisation and a sliding mode controller are employed, and the superiority of the robust one is proved since the cancer model is affected by biologic parametric uncertainties and environmental disturbances. Two numerical optimisation algorithms are also used to optimise the therapy parameters such as hyperthermia and improve the efficiency of chemotherapy, including the genetic algorithm and fuzzy tuner, and their related results are compared. This optimisation is necessary to decrease the settling time as the treatment duration and also decrease the response overshoot as the injection side effects. As a result, an optimal robust controller is designed in this paper for the presented model of STR Pinho. It is shown that using the proposed optimal robust controller, chemotherapy can be elaborated successfully with the least amount of side effects and lower affectability from the biological parametric uncertainties, and the best treatment efficiency within a finite day can be achieved. This paper is organised as follows: In Section 2, the mathematical model of the cancer is presented. The controller design based on the two mentioned controlling methods using anti‐angiogenic is explained in Section 3. Afterwards, in Section 4, the optimisation processes of Genetic Algorithm (GA) and fuzzy are mentioned. Comparative results and verification process of the proposed algorithms are conducted in Section 5 with the aid of some simulation scenarios conducted in MATLAB and comparing the results with previous studies.

## 2 MATHEMATICAL MODEL

Considering the fact that the dynamics of different types of cancer for different tissues of the body are significantly different, here in this section, the analytic model of the cancer dynamic related to vascular tumours of dermal tissue is extracted as the case study of the implementation of the proposed controller considering the anti‐angiogenic input. The proposed model is related to neoplastic disease and models the vascular tumour growth for which the number of the cells is very large and the tumour diameter is *D* > 2 mm [7]. This treatment is also useful in colorectal tumours based on Ref. [29]. The base model, which is employed here is verified in Ref. [7], and its correctness is proved by comparing the analytical results with numerical ones. In this reference, it is proved that this mentioned state space can describe the growth of the vascular tumours related to the dermal cancers. This model shows the unstable condition of the cancerous cells in the absence of the chemotherapy drug. Moreover, the effect of the anti‐angiogenic input on the cells' growth is obviously traceable in this model. At first, the anti‐angiogenic drugs and their related effects on cancer treatment are discussed. Angiogenesis is the natural phenomenon in the body, and its duty is to define the production of blood vessels for the growth and repair of the tissues in the body. Vasudev et al. demonstrated that when the size of the tumour is bigger than 3 mm, the tumour releases signals that cause the endothelial cells to provide nutrients and oxygen for the survival of the cancer cells [32]. It means there is a battle between the normal and cancer cells for using the nutrients and oxygen in the blood [33]. The winner of this battle is unfortunately the cancer cells.

Anti‐angiogenic is one of the drug control methods in which the amount of the drug is decreased continuously, but it will not be stopped. It should be noted that when one stops the procedure suddenly, the procedure of transferring the nutrients will be blocked for both of the cancer cells as the same as the normal cells. Thus, this process results in death. In other words, the anti‐angiogenic drug makes the cells hungry and weak during chemotherapy. It is worth mentioning that the anti‐angiogenic drug cannot destroy the cancer cells as the sole target. However, if one uses fewer chemotherapy drugs in a controlled way, then there is less damage to the body and the normal cells. There are five state variables in the system dynamic proposed by Pinho et al. [7]: normal cells (NCs), cancer cells (CCs), endothelial cells (ECs), chemotherapy agents (CA), and anti‐angiogenic agents (AA) in the body, which are indicated by $x_{1}$, $x_{2}$,$x_{3}$, y, and w, respectively. In this section, only the non‐dimensional dynamic is written, which is shown in Equation ((1a), (1b), (1c), (1d), (1e)).

(1a) $$\frac{dx_{1}}{dt}=\alpha_{1}x_{1}\left(t\right)\left(1-x_{1}\left(t\right)\right)-q_{1}x_{1}\left(t\right)x_{2}\left(t\right)-p_{1}\left(x_{3}\left(t\right).w\left(t\right)\right)\frac{x_{1}\left(t\right)y\left(t\right)}{a_{1}+x_{1}\left(t\right)}$$

(1b) $$\frac{dx_{2}}{dt}=\alpha_{2}x_{2}\left(t\right)\left[1-\frac{x_{2}\left(t\right)}{1+\gamma x_{3}\left(t\right)}\right]-q_{2}x_{1}\left(t\right)x_{2}\left(t\right)-p_{2}\left(x_{3}\left(t\right).w\left(t\right)\right)\frac{x_{2}\left(t\right)y\left(t\right)}{a_{2}+x_{2}\left(t\right)}$$

(1c) $$\frac{dx_{3}}{dt}=\beta x_{2}\left(t\right)+\alpha_{3}x_{3}\left(t\right)\left[1-x_{3}\left(t\right)\right]-\frac{p_{3}x_{3}\left(t\right)w\left(t\right)}{a_{3}+x_{3}\left(t\right)}$$

(1d) $$\frac{dy}{dt}=\delta -\left[\xi +d_{1}\frac{x_{1}\left(t\right)}{\alpha_{1}+x_{1}\left(t\right)}+d_{2}\frac{x_{2}\left(t\right)}{a_{2}+x_{2}\left(t\right)}\right]y\left(t\right)$$

(1e) $$\frac{dw}{dt}=\phi -\left[\eta +\frac{d_{3}x_{3}\left(t\right)}{\alpha_{3}+x_{3}\left(t\right)}\right]w\left(t\right)$$

Here $\alpha_{1}$, $\alpha_{2}$, $\alpha_{3}$ are NCs, CCs and ECs proliferation rate, respectively, ξ is the chemical washout rate, and η is the anti‐angiogenic washout rate. $a_{1}$,$a_{2}$ and $a_{3}$ are the saturation rate on NCs, CCs and ECs, respectively, $q_{1}$ and $q_{2}$ are competitive rate of NCs and CCs, β is the CCs production rate due to ECs and γ is the coefficient of ECs for blood supply to the tumour. Also $p_{i}\left(x_{3}\left(t\right)∙w\left(t\right)\right)$ represents $p_{i0}+p_{i1}x_{3}\left(t\right)+p_{i2}w\left(t\right)$ for $i=1.2.3\;and\;x_{i}\left(0\right)\geq 0$. Moreover, $p_{10}$ is NCs loss rate by CA and $p_{11}$ is the rate of ECs cooperation on CA for NCs, $p_{12}$ is the rate of AA cooperation on CA for NCs, $p_{20}$ represents the CCs loss rate by CA, $p_{21}$ is the rate of ECs cooperation on CA for CCs, $p_{22}$ is the rate of AA cooperation on CA for CCs and $p_{3}$ is the ECs loss rate by AA. Finally, $p_{i0}$, *i* = 1, 2, is the killing rate of chemotherapy on $x_{i}$ in the absence of $x_{3}$ and *w*, respectively; $p_{ij}$, *i*, *j* = 1, 2, is the increased rate of killing of $x_{i}$ by CA per concentration of $x_{3}$ (*j* = 1) and *w* (*j* = 2).

Also note that here *w* is a function of only *x*_3_ [7]. The reason is attributed to the fact that here the anti‐angiogenic cannot improve cancer by itself, and it just decreases the feeding of cancerous cells by reducing the endothelial cells. Thus, this goal will be fulfiled by controlling the endothelial cells. As a result, it can be concluded that in the dynamics of *w*, the significant term is *x*_3_ and the other parameters, that is, *x*_1_ and *x*_2_ do not play an important role in the mentioned target. In this model, the tumour cells and non‐tumour cells interact with each other since according to Equations (1a) and (1b), the related states are coupled. The interaction between the mentioned cells is in a way that each type tries to reduce the number of the other types. The rate of this interaction depends on the values $q_{1}.q_{2}$. The numerical values of the mentioned parameters are described in Table 4 according to Ref. [7]

The normalised values and the concept of each coefficient are mentioned in Ref. [21]. The assumptions of the mentioned system model are as follows:

A) As long as the cell growth is speedy and their number is very large and uncountable, the sample rate is satisfactory, and thus the model can be considered as a continuous model. Thus, the governing equations are ODEs in which the states vary with time.
B) There is a homogeneous distribution of the cells in the body tissues affected by the drugs. The CA cannot destroy just the cancer cells. In other words, it eliminates both the NCs and CCs with different rates.
C) The effect of the CA on EC can be ignored, and it means that the EC is decoupled from CA.
D) $x_{1}$ and $x_{2}$ grow logistically and compete for living. $x_{3}$ also grows, however, its development rate is less than two previous states.
E) CA acts as a fatal agent with different coefficients on NCs and CCs. AA only eliminates ECs.
F) These terms have appeared in the form of a saturation function relative to $x_{i}$ in the presented ODEs. In other words, the maximum effect of the drug on the cells is one as $x_{i}$ diverge to infinity.
G) y and w have a direct relation with the continuous injection rate of each drug (incremental factor).
H) Due to the half‐life of drugs, medications will be lost in the patient's body. Also, cells in the body kill them (decrement factors).

Note that one of the superiorities of the proposed model is its analytic nature for which all of the state variables are always in the positive region since the initial conditions are non‐negative [7]. As was mentioned, both the CA and AA can destroy the CCs. Now four situations can be supposed:

Situation 1—No Cure:

When no drug is employed, the winner of the battle is CCs, according to experimental results, and it means that the body immune system is not strong enough to cope with the disease. This condition usually results in the death of the patient at last.

Situation 2—No CA:

In this condition, y=0 and from the first and second equations, it can be concluded that the condition is the same as Situation 1. Consequently, the CCs win the battle again. This is attributed to the fact that the AAs cannot destroy the CCs by their own, and they can just prevent them from using nutrients. This phenomenon is verifiable by experiment again.

Situation 3—No AA:

It can be concluded from the theoretical concepts that cures can be realised in this situation by CA only. In order to investigate the stability of the equilibrium point (if exists) in this situation, the non‐linear system should be linearised around this point. But, for destroying the CCs by just CA, more drug is required. Also considering the fact that CA also hurts the NCs, it is not a good idea to use CA without AA.

Situation 4—CA and AA:

As discussed, the best way is the simultaneous usage of CA and AA for the cure. As shown in this paper, AA helps CA destroy the CCs with fewer amount of drugs while the response is also faster. This method with less CA has minimum casualties for the patient. This type of cancer is called Chemo‐Resistant tumours. This point was derived from laboratory results reported by Browder et al. and indicated the validity of this model [29]. Also CA and AA is recommended in the case of colorectal tumours [34].

Thus, the conclusions can be summarised as follows:

- CCs cannot be killed by AA only.
- In the case of a Chemo‐Resistant tumour, CA cannot cure the patient individually. But in other cases, CA may eliminate the CCs under some specific conditions.
- The cooperation between CA and AA is the most effective way of reducing the CCs compared to the sole CA.

## 3 CONTROLLER DESIGN

As mentioned the mathematical model of cancer is opted here from Ref. [7] and the main contribution of the paper is to design and implement an efficient closed loop robust controller in which the gains are tuned in an adaptive way using fuzzy algorithm and is optimised using genetic algorithm tool simultaneously. Also anti‐angiogenic is proposed here to reduce the side effects of the suggested therapy.

In this section, at first, an Input–Output Feedback Linearisation (IOFL) controller is designed for CA, and the results are investigated for the case in which no AA is employed. Afterwards, a controller is designed for AA, and both of the CA and AA are applied on the system simultaneously. Finally, the robustness of the system in the presence of the body parametric uncertainties is increased using the Sliding Mode Controller. The results are compared and analysed. In the next section, genetic algorithm and fuzzy tuner are employed to optimally gain tuning in and to find the best method to destroy CCs with higher speed and also to use less amount of CA.

### 3.1 Feedback linearisation controlling method

Here the aim is to control the cancer cells in a closed loop way using a proper non‐linear controller. Feedback linearisation and SMC are among the most popular and applicable closed loop non‐linear controllers, which are employed in this paper. However, since all of the states are not important for us (just the cancerous cells need to be controlled while the feedback and controlling the healthy ones are not necessary) the Input‐Output Feedback Linearisation method is used instead of Input State one. Since the main goal is minimising the cancer cells, the output of the system is considered as $x_{2}$, which is the number of the cancer cells.

Part 1

Here, the main goal is to destroy CCs and decrease them to zero, so the output is set as $x_{2}$ and a regulator controller is provided to set the output on zero. In the IOFL method, sequential derivation of the output should be conducted till the input appears. With the aid of the input, one can linearise the equation and design a controller for the linear system. We have:

(2) $$x_{2}=O$$

where *O* is output. Thus,

(3) $$\frac{\partial }{\partial t}x_{2}=\dot{O}={\dot{x}}_{2}=\alpha_{2}x_{2}-\frac{\alpha_{2}x_{2}^{2}}{1+\gamma x_{3}}-q_{2}x_{1}x_{2}-p_{2}\left(x_{3}.w\right)\frac{x_{2}y}{a_{2}+x_{2}}$$

(4) $$\ddot{O}=\alpha_{2}{\dot{x}}_{2}-\frac{2\alpha_{2}{\dot{x}}_{2}x_{2}\left(1+\gamma x_{3}\right)-\alpha_{2}x_{2}^{2}\gamma {\dot{x}}_{3}}{{\left(1+\gamma x_{3}\right)}^{2}}-q_{2}{\dot{x}}_{1}x_{2}-q_{2}x_{1}{\dot{x}}_{2}-\left(p_{21}{\dot{x}}_{3}+p_{22}\dot{w}\right)\frac{x_{2}y}{a_{2}+x_{2}}$$

Input signal is appeared in the second derivation of the output. Substituting $\dot{y}$ in Equation (1d) we have:

(5) $$\begin{array}{l} \ddot{O}=\alpha_{2}{\dot{x}}_{2}-\frac{2\alpha_{2}{\dot{x}}_{2}x_{2}\left(1+\gamma x_{3}\right)-\alpha_{2}x_{2}^{2}\gamma {\dot{x}}_{3}}{{\left(1+\gamma x_{3}\right)}^{2}}-q_{2}{\dot{x}}_{1}x_{2}-q_{2}x_{1}{\dot{x}}_{2}-\left(p_{21}{\dot{x}}_{3}+p_{22}\dot{w}\right)\frac{x_{2}y}{a_{2}+x_{2}}\\ -p_{2}\left(x_{3}.w\right)\frac{{\dot{x}}_{2}y\left(x_{2}+x_{2}\right)-x_{2}{\dot{x}}_{2}y}{{\left(a_{2}+x_{2}\right)}^{2}}-\frac{p_{2}\left(x_{3}.w\right)}{{\left(a_{2}+x_{2}\right)}^{2}}\times \left(a_{2}+x_{2}\right)x_{2}\left(u-\left[\xi +\frac{d_{1}x_{1}}{a_{1}+x_{1}}+\frac{d_{2}x_{2}}{a_{2}+x_{2}}\right]y\right) \end{array}$$

Implementing more simplification results in

(6) $$\begin{array}{l} \ddot{O}=\alpha_{2}{\dot{x}}_{2}-\frac{2\alpha_{2}{\dot{x}}_{2}x_{2}\left(1+\gamma x_{3}\right)-\alpha_{2}x_{2}^{2}\gamma {\dot{x}}_{3}}{{\left(1+\gamma x_{3}\right)}^{2}}-q_{2}{\dot{x}}_{1}x_{2}-q_{2}x_{1}{\dot{x}}_{2}-\left(p_{21}{\dot{x}}_{3}+p_{22}\dot{w}\right)\frac{x_{2}y}{a_{2}+x_{2}}\\ -p_{2}\left(x_{3}.w\right)\frac{{\dot{x}}_{2}y\left(x_{2}+x_{2}\right)-x_{2}{\dot{x}}_{2}y}{{\left(a_{2}+x_{2}\right)}^{2}}-\frac{p_{2}\left(x_{3}.w\right)}{\left(a_{2}+x_{2}\right)}\times x_{2}\left(u-\left[\xi +\frac{d_{1}x_{1}}{a_{1}+x_{1}}+\frac{d_{2}x_{2}}{a_{2}+x_{2}}\right]y\right) \end{array}$$

And now it is possible to extract the input in a way that the equation could be linearised:

(7) $$\begin{array}{l} u=\left[\xi +\frac{d_{1}x_{1}}{a_{1}+x_{1}}+\frac{d_{2}x_{2}}{a_{2}+x_{2}}\right]y+\frac{a_{2}+x_{2}}{p_{2}\left(x_{3}\cdot w\right)\times x_{2}}\{-p_{2}\left(x_{2}\cdot w\right)\frac{{\dot{x}}_{2}y\left(a_{2}+x_{2}\right)-x_{2}{\dot{x}}_{2}y}{{\left(a_{2}+x_{2}\right)}^{2}}-\alpha_{2}{\dot{x}}_{2}\\ +\frac{2\alpha_{2}{\dot{x}}_{2}x_{2}\left(1+\gamma x_{3}\right)-\alpha_{2}x_{2}^{2}\gamma {\dot{x}}_{3}}{{\left(1+\gamma x_{2}\right)}^{2}}q_{2}{\dot{x}}_{1}x_{2}+q_{2}x_{1}{\dot{x}}_{2}+\left(p_{21}{\dot{x}}_{3}+p_{22}\dot{w}\right)\frac{x_{2}y}{a_{2}+x_{2}}+\nu \} \end{array}$$

Here *v* is defined as follows to provide the possibility of pole placement:

(8) $$\nu =-k_{11}{\dot{x}}_{2}-k_{10}x_{2}$$

Thus, the linearised system can be defined as follows in which the pole placement can be implemented:

(9) $$z_{1}=x_{2}\cdot z_{2}={\dot{x}}_{2}$$

(10) $$\left\{\begin{array}{l} {\dot{z}}_{1}=z_{2}\\ {\dot{z}}_{2}=-k_{10}z_{1}-k_{11}z_{2} \end{array}\right.$$

Part 2

Here AA controller is supposed to be designed. To meet this goal, it is desired to decrease $x_{3}$ to the smallest possible value but not zero. It should be noted that with the aid of gain tuning it is possible to make $x_{3}$ small but not zero. So, we define $x_{3}$as the output:

(11) $$Q=x_{3}\to \dot{Q}={\dot{x}}_{3}=\beta x_{3}\left(t\right)+\alpha_{3}x_{3}\left(t\right)\left[1-x_{3}\left(t\right)\right]-\frac{p_{3}x_{3}\left(t\right)w\left(t\right)}{a_{3}+x_{3}\left(t\right)}$$

(12) $$\begin{array}{l} \ddot{Q}=\beta {\dot{x}}_{3}+\alpha_{3}{\dot{x}}_{3}-2\alpha_{3}{\dot{x}}_{3}\\ -\frac{\left(p_{3}{\dot{x}}_{3}\left(t\right)w\left(t\right)+p_{3}x_{3}\left(t\right)\dot{w}\left(t\right)\right)\left(a_{3}+x_{3}\left(t\right)\right)-{\dot{x}}_{3}\left(t\right)\left(p_{3}x_{3}a_{3}+x_{3}\left(t\right)wa_{3}+x_{3}\left(t\right)\right)}{{\left(a_{3}+x_{3}\left(t\right)\right)}^{2}} \end{array}$$

Also considering Equation (1) the input signal is

(13) $$\begin{array}{l} \phi =\left[\eta +\frac{d_{3}x_{3}\left(t\right)}{a_{3}+x_{3}\left(t\right)}\right]w\left(t\right)\\ -\frac{1}{p_{3}x_{3}\left(t\right)}\left(p_{3}{\dot{x}}_{3}\left(t\right)w\left(t\right)-\frac{1}{a_{3}+x_{3}\left(t\right)}\left(p_{3}{\dot{x}}_{3}w\left(t\right)x_{3}\left(t\right)\right)-{\left(a_{3}+x_{3}\left(t\right)\right)}^{2}\left(\beta {\dot{x}}_{3}+\alpha_{3}{\dot{x}}_{3}-2\alpha_{3}{\dot{x}}_{3}+\nu \right)\right) \end{array}$$

And *v* is given as bellow:

(14) $$\nu =-k_{21}{\dot{x}}_{3}-k_{20}x_{3}$$

Again here the linearised system can be defined as follows:

(15) $$q_{1}=x_{3}\cdot q_{2}={\dot{x}}_{3}$$

(16) $$\left\{\begin{array}{l} {\dot{q}}_{1}=q_{2}\\ q_{2}=-k_{20}q_{1}-k_{21}q_{2} \end{array}\right.$$

### 3.2 Sliding mode controlling method

It should be considered that, in biological dynamic systems, the parameters cannot be defined perfectly and there are always a lot of parametric uncertainties. The main sources of the mentioned uncertainties are due to either the lack of information about the body or the differences in the body's reaction to the external signals for different patients. For example, we know that the effect of the chemotherapy drug or anti‐angiogenic on the cancer cells is not similar in all cases. The other example is related to the growth rate of the cancer and normal cells, which is not unique for all the patients. As a result, the necessity of a robust non‐linear control is unavoidable to compensate the mentioned uncertainties and provide the stability of the treatment for all of the case studies. Thus, the strong robust control of sliding mode is designed to inject the chemotherapy drug and its related anti‐angiogenic. The first step is to define a proper sliding surface ‘s’ according to the states of the system:

(17) $$s_{1}={\dot{e}}_{1}+k_{1}e_{1}$$

(18) $$e_{1}=x_{2}-x_{2d}$$

where $e_{1}$ is the error signal and $x_{2d}$ is the desired value of the variable $x_{2}$, which should be considered zero for this system to vanish the cancer cells.

The related Lyapunov function is considered as follows, which is positive definite:

(19) $$V_{1}=\frac{1}{2}s_{1}^{2}$$

Derivation of the mentioned Lyapunov candidate with respect to time results in

(20) $$\begin{array}{l} {\dot{V}}_{1}=s_{1}\left({\ddot{e}}_{1}+k_{1}{\dot{e}}_{1}\right)=s_{1}\{\alpha_{2}{\dot{x}}_{2}-\frac{\left(2\alpha_{2}x_{2}{\dot{x}}_{2}\right)\left(1+\gamma x_{3}\right)-\left(\alpha_{2}x_{2}^{2}\gamma {\dot{x}}_{3}\right)}{{\left(1+\gamma x_{3}\right)}^{2}}-q_{2}\left({\dot{x}}_{1}x_{2}+x_{1}{\dot{x}}_{2}\right)\\ -\left(p_{21}{\dot{x}}_{3}+p_{22}\dot{w}\right)\frac{x_{2}y}{a_{2}+x_{2}}-\left(p_{20}+p_{21}x_{3}+p_{22}w\right)\frac{\left({\dot{x}}_{2}y+x_{2}\dot{y}\right)\left(a_{2}+x_{2}\right)-\left(x_{2}{\dot{x}}_{2}y\right)}{{\left(a_{2}+x_{2}\right)}^{2}}+k_{1}{\dot{x}}_{2}\} \end{array}$$

which is negative definite; here, by substituting $\dot{y}$, the controlling signal of chemotherapy injection appears that can be seen as follows:

(21) $$\begin{array}{l} \delta =\left(\xi +d_{1}\frac{x_{1}}{a_{1}+x_{1}}+d_{2}\frac{x_{2}}{a_{2}+x_{2}}\right)y-\frac{a_{2}+x_{2}}{\left(p_{20}+p_{21}x_{3}+p_{22}w\right)x_{2}}\{\left(p_{20}+p_{21}x_{3}+p_{22}w\right)\\ \;\frac{\left({\dot{x}}_{2}y\right)\left(a_{2}+x_{2}\right)-\left(x_{2}{\dot{x}}_{2}y\right)}{{\left(a_{2}+x_{2}\right)}^{2}}+q_{2}\left({\dot{x}}_{1}x_{2}+x_{1}{\dot{x}}_{2}\right)-\frac{\left(2\alpha_{2}x_{2}{\dot{x}}_{2}\right)\left(1+\gamma x_{3}\right)-\left(\alpha_{2}x_{2}^{2}\gamma {\dot{x}}_{3}\right)}{{\left(1+\gamma x_{3}\right)}^{2}}\\ +\alpha_{2}{\dot{x}}_{2}-k_{2}\;\tan^{-1}\left(s\right)\} \end{array}$$

Here in order to avoid the chattering phenomenon, ‘$\tan^{-1}\left(s\right)$’ is used for switching rather than ‘sign (s)’. The same procedure can also be done for the anti‐angiogenic input by defining the following sliding surface:

(22) $$s_{2}={\dot{e}}_{1}+k_{3}e_{1}$$

where $e_{1}$ is the same as Equation (18). Now by considering the Lyapunov function in Equation (19), its related derivation can be extracted as given in Equation (24).

(23) $$V_{2}=\frac{1}{2}s_{2}^{2}$$

(24) $$\begin{array}{l} {\dot{V}}_{2}=s_{2}\left({\ddot{e}}_{1}+k_{3}{\dot{e}}_{1}\right)=s_{2}\{\alpha_{2}{\dot{x}}_{2}-\frac{\left(2\alpha_{2}x_{2}{\dot{x}}_{2}\right)\left(1+\gamma x_{3}\right)-\left(\alpha_{2}x_{2}^{2}\gamma {\dot{x}}_{3}\right)}{{\left(1+\gamma x_{3}\right)}^{2}}-q_{2}\left({\dot{x}}_{1}x_{2}+x_{1}{\dot{x}}_{2}\right)\\ -\left(p_{21}{\dot{x}}_{3}+p_{22}\dot{w}\right)\frac{x_{2y}}{a_{2}+x_{2}}-\left(p_{20}+p_{21}x_{3}+p_{22}w\right)\frac{\left({\dot{x}}_{2}y+x_{2}\dot{y}\right)\left(a_{2}+x_{2}\right)-\left(x_{2}{\dot{x}}_{2}y\right)}{{\left(a_{2}+x_{2}\right)}^{2}}k_{3}{\dot{x}}_{2}\} \end{array}$$

Here again considering the Equation (1e) and by substitution of $\dot{w}$ the related controlling signal can be extracted accordingly:

(25) $$\begin{array}{l} \varphi =\left(\eta +\frac{d_{3}x_{3}}{a_{3}+x_{3}}\right)w+\frac{1}{p_{22}}\left[-p_{21}{\dot{x}}_{3}-\frac{a_{2}+x_{2}}{x_{2}y}\{q_{2}\left({\dot{x}}_{1}x_{2}+x_{1}{\dot{x}}_{2}\right)-\alpha_{2}{\dot{x}}_{2}\right]\\ +\frac{\left(2\alpha_{2}x_{2}{\dot{x}}_{2}\right)\left(1+\gamma x_{3}\right)-\left(\alpha_{2}x_{2}^{2}\gamma {\dot{x}}_{3}\right)}{{\left(1+\gamma x_{3}\right)}^{2}}-\left(p_{20}+p_{21}x_{3}+p_{22}w\right)\frac{\left(x_{2}{\dot{x}}_{2}y\right)}{{\left(a_{2}+x_{2}\right)}^{2}}-k_{3}{\dot{x}}_{2}-k_{4}\;\tan^{-1}\left(s\right)\} \end{array}$$

It should be noted that according to Ref. [7], it is supposed that the distribution of the cells in a single tissue is roughly homogeneous in a way that it can be assumed that the treatment input acts on every cell's pack. Here, the employed controlling gains should be determined so that the best efficiency of the cancer cell destruction could be realised with the least probable side effects.

## 4 GAIN TUNING AND OPTIMISATION

Here two numerical methods of GA and Fuzzy tuning are employed to optimise the gains of the designed feedback linearisation controller. These algorithms are of the strongest and most popular numerical optimisation methods.

### 4.1 The genetic algorithm optimisation tool

Here an objective function should be defined and a gene is defined according to these parameters. The values of the parameters of this gene change internationally and the objective function is updated in each iteration. The changes of the gene parameters mutes such as the human gene and some biologically inspired algorithms will be employed to find the optimum values of the parameters in a way the objective function would set on its extremum value. The implementation of this algorithm in each computational iteration is performed by three main operands, including two genetic operations and one randomly selected one. Since the target of control is a biological parameter and follows the biological behaviours, selecting this algorithm increases the optimisation process of controlling cancer.

Here six parameters of FL are engaged in the GA process, including $k_{10}$ and $k_{11}$ related to the chemotherapy, $k_{20}$ and $k_{21}$ related to anti‐angiogenic and the two last ones are the gain of the two controllers. The cost function is considered as below:

(26) $$\mathrm{Cos}t=\sum \left(x_{2}^{2}\right)V_{1}+\sum \left(y^{2}\right)V_{2}+\sum \left({\left(x_{3}-0.05\right)}^{2}\right)V_{3}+\sum \left(w^{2}\right)V_{4}$$

where $V_{i}\forall i\in 1,2,3,4$ are weighting gains of the cost function and are considered as follows:

(27) $$V_{1}=500V_{2}=V_{3}=500V_{4}$$

**FIGURE 1:**
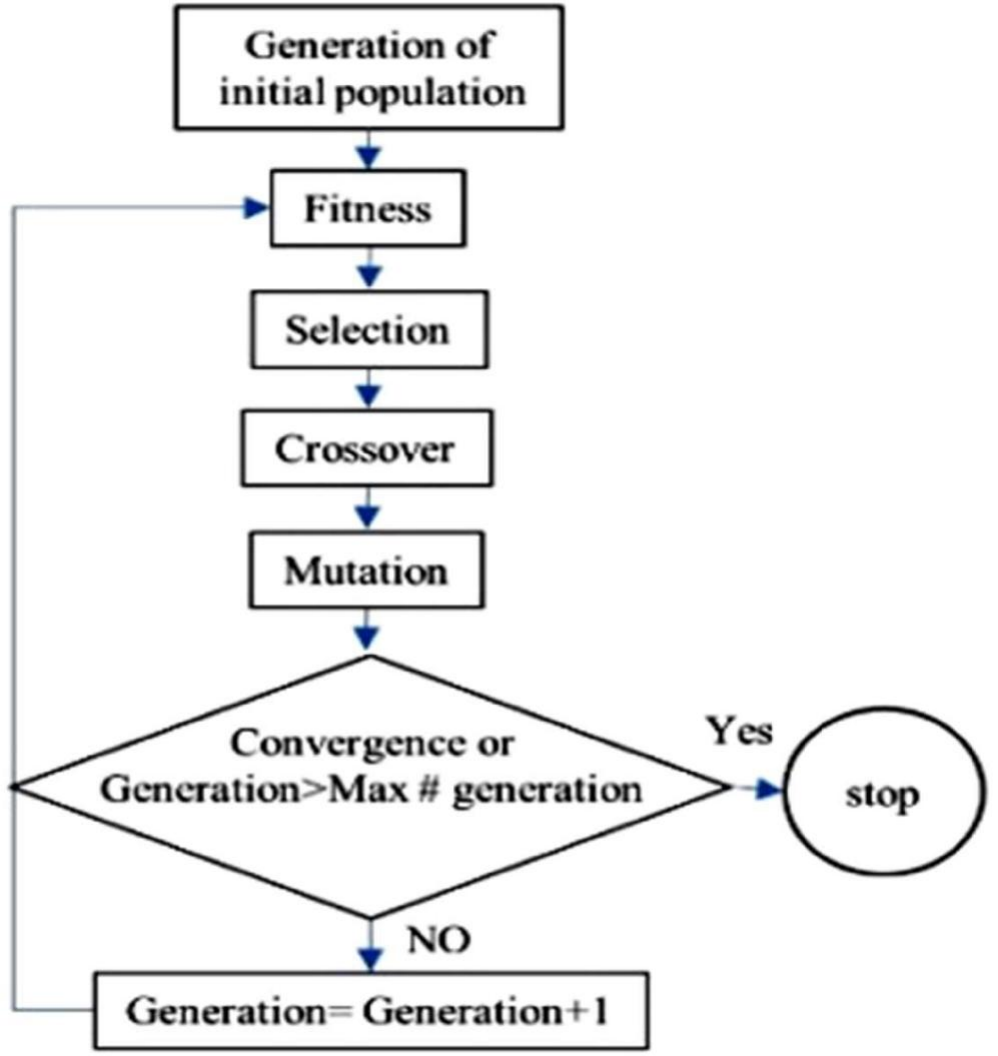
Genetic algorithm chart

The process of gain tuning according to the GA for the designed FL controller is depicted in Figure 1 as the following flowchart:

Here our main goal is to kill all cancer cells with a minimum chemotherapy drug. Also, for further efficiency of the chemotherapy drug, it is required to minimise the endothelial cells, and this target is fulfiled here using anti‐angiogenic. Considering the fact that endothelial cells are responsible for feeding both of the normal and cancer cells, their decrease to zero is dangerous and it results in the death of the normal cells. That's why at the above‐mentioned cost function the desired value of these cells is set as 0.05, which provides a compromise between weakening the cancer cells and also feeding the normal ones simultaneously.

The extracted optimum values of both the FL and SMC controllers based on the genetic optimisation tool are as given in Table 1:

**TABLE 1:** The extracted optimum controlling gains with the aid of a genetic algorithm

Sliding mode control gains		Feedback linearisation gains	
$k_{1}$	3.00	$k_{10}$	−0.018
$k_{2}$	3.00	$k_{11}$	−0.029
$k_{3}$	0.05	$k_{20}$	−2.059
$k_{4}$	0.05	$k_{21}$	−2.500

### 4.2 Fuzzy tuning optimisation tool

**FIGURE 2:**
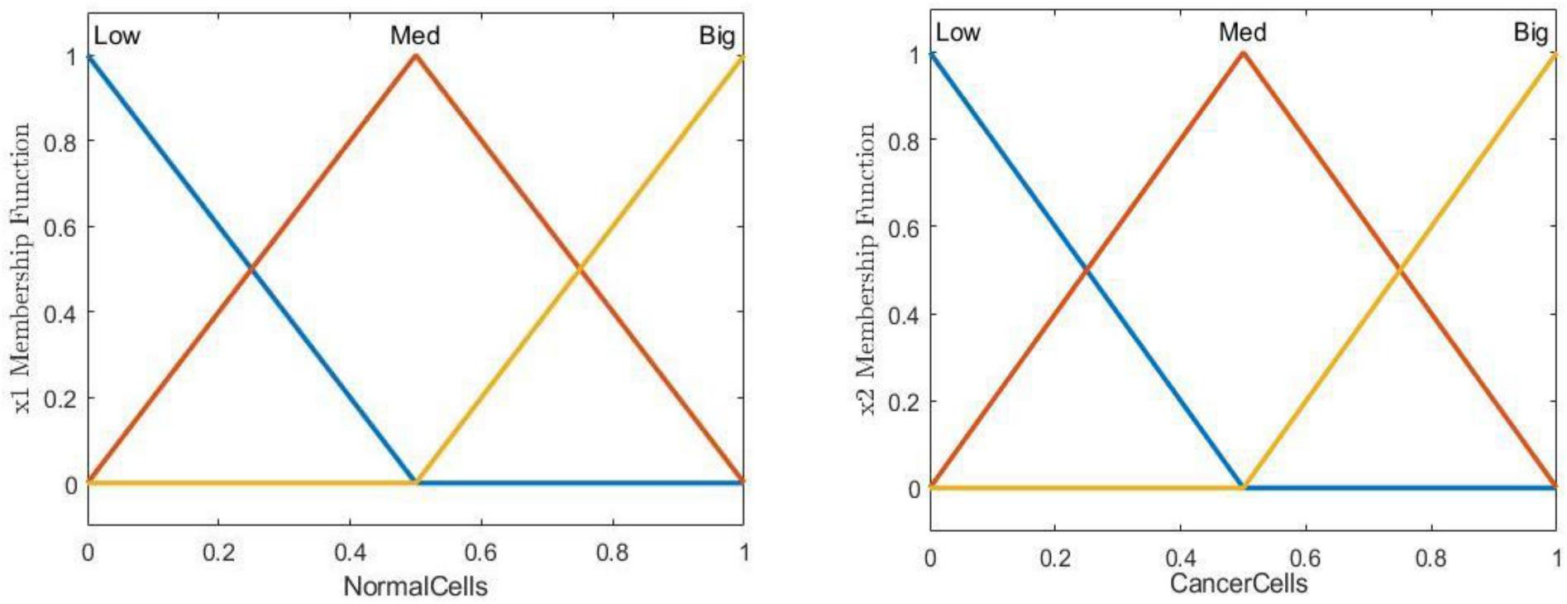
The curve of the considered membership functions of cancerous and normal cells

**FIGURE 3:**
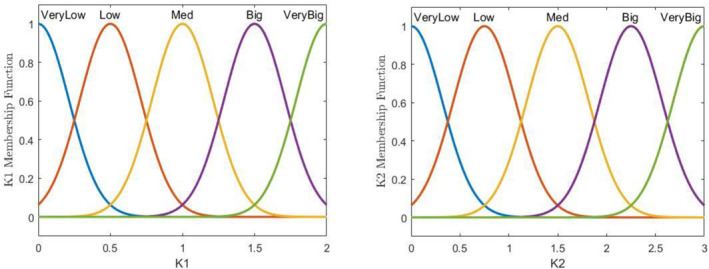
The output membership functions of controlling gains ${K_{2}∙K}_{1}$

**FIGURE 4:**
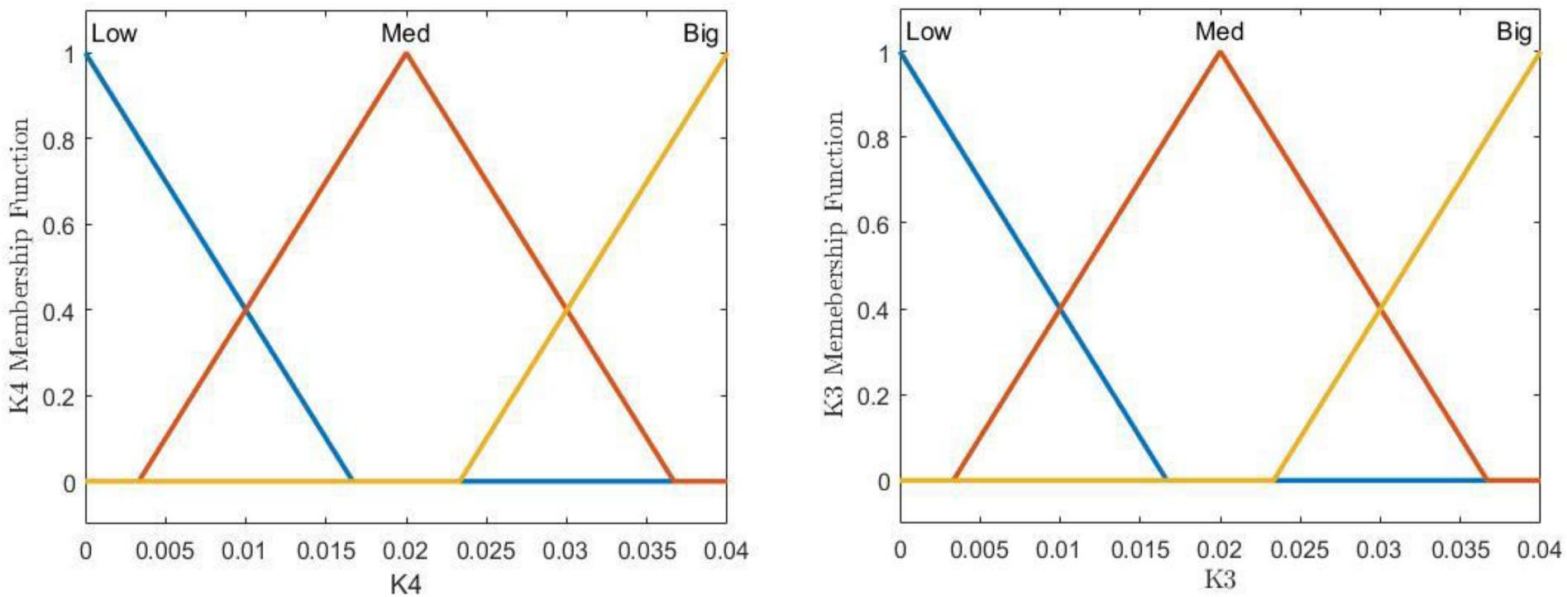
The output membership functions of controlling gains ${K_{4}∙K}_{3}$

Since the performance of the sliding mode controller is extremely dependent on its related controlling gains, its related gains are also optimised using an alternative optimisation tool that is fuzzy tuner. The gains employed in Section 3 are tuned manually and are also constant. Thus, considering the fact that the general condition of the patient is time dependent, it can be concluded that these gains are not condition efficient. With the aid of the proposed fuzzy tuning algorithm, it is possible to set the gains as a function of the instantaneous number of the cancerous and normal cells. As a result, the treatment duration and its corresponding side effects will be decreased enormously. In order to establish the fuzzy tuner module, the number of the cancerous and normal cells are employed as the system input and using the fuzzy rules and memberships, the proper controlling gains can be extracted as the output. The triangular membership functions are defined for state *x*1 (healthy cells) and for state *x*2 (cancer cells) as illustrated in Figure 2. Also for the outputs, membership functions of control gains *k*1 and *k*2 are defined according to Gaussian functions, which are shown in Figure 3 and the system output membership functions for control gains *k*3 and *k*4 are represented in Figure 4.

Here the related controlling gains are also defined as a Gaussian shape as follows

The employed fuzzy motor in this paper is Mamdani system, in which the output can be evaluated using Equation (28):

(28) $$K_{j}=\frac{\sum_{i=1}^{9}\alpha_{i}r_{i}}{\sum_{i=1}^{9}r_{i}}\forall j\in 1,2,3,4$$

Here $\alpha_{i}$is the coefficient of fire, which is within 0 and 1 depending on the number of the cancerous and normal cells. $r_{i}$ is the membership function value of the output of each rule. The considered rules of the established fuzzy system for simple chemotherapy and the one which is modified by the anti‐angiogenic are mentioned in Tables 2 and 3.

**TABLE 2:** The rules of controlling gains of the chemotherapy input

		Cancer cell ($x_{2}$)		
$K_{1}$		Low	Medium	Big
Normal cells ($x_{1}$)	Low	Med.	Big	Very big
	Medium	Med.	Med.	Big
	Big	Very low	Med.	Big

		Cancer cell ($x_{2}$)		
$K_{2}$		Low	Medium	Big
Normal cells ($x_{1}$)	Low	Low	Big	Very big
	Medium	Low	Big	Very big
	Big	Very low	Med.	Big

**TABLE 3:** The rules of controlling gains of anti‐angiogenic input

		Cancer cell ($x_{2}$)		
$K_{3}$		Low	Medium	Big
Normal cells ($x_{1}$)	Low	Med.	Big	Big
	Medium	Med.	Med.	Big
	Big	Low	Low	Med.

		Cancer cell ($x_{2}$)		
$K_{4}$		Low	Medium	Big
Normal cells ($x_{1}$)	Low	Med.	Med.	Big
	Medium	Med.	Med.	Big
	Big	Low	Low	Med.

**FIGURE 5:**
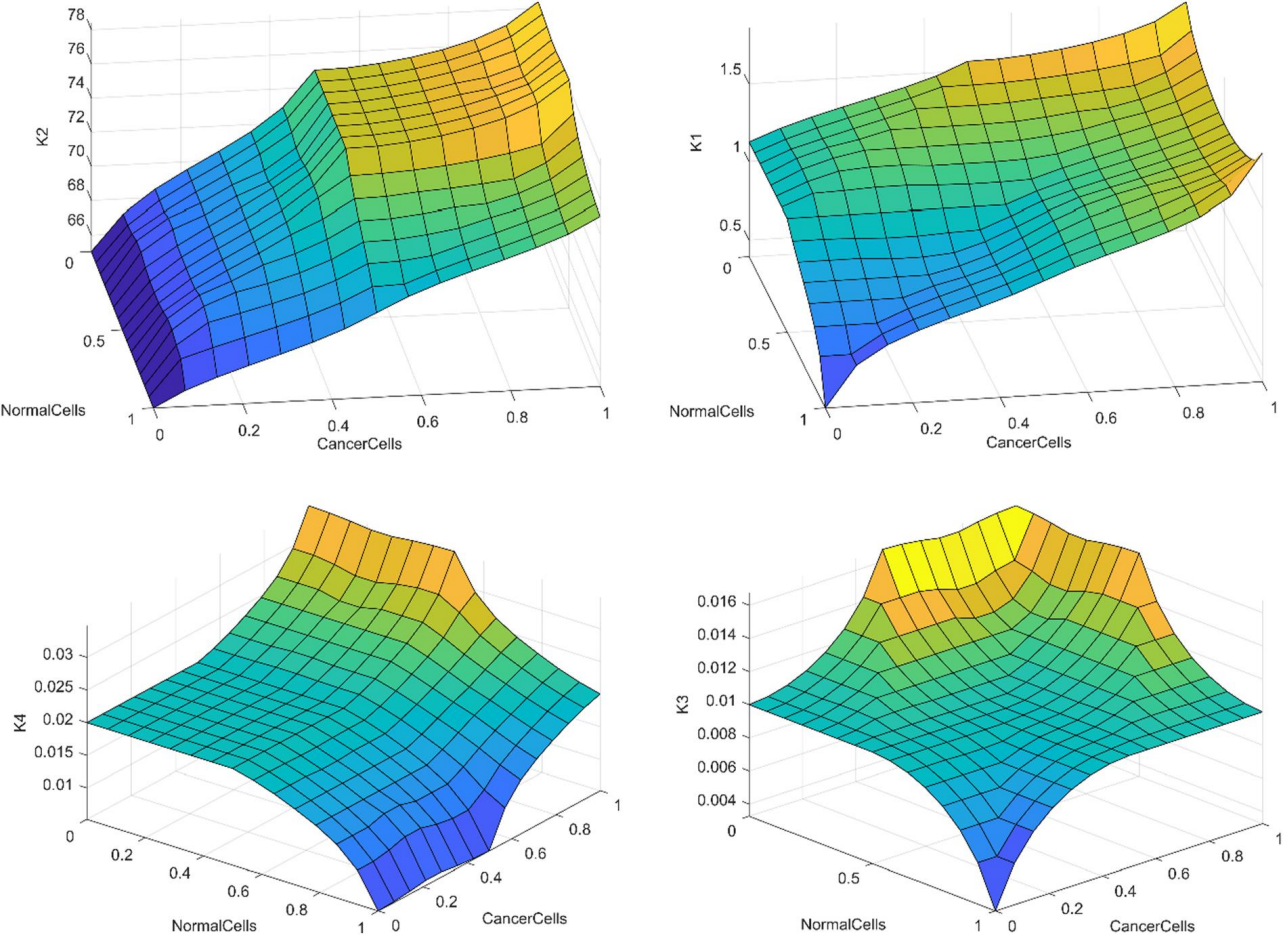
The fuzzy rule surfaces

About the controlling gain related to the anti‐angiogenic input special consideration is required since its additional decrease will result in increasing the cancerous cells and its additional increase will destroy the endothelial cells and the death of the patient. The surfaces of the considered fuzzy rules are shown in Figure 5:

As can be seen, the fuzzy rules are defined so that the resulting controlling gains have a direct relation with the normal cells and an inverse relation with the cancer ones. As a result, the chemotherapy input will be decreased as a result of decreasing the cancer cells, which consequently results in lower side effects.

## 5 SIMULATION STUDY AND VERIFICATION

The efficiency of the proposed controller and its related optimisation process are investigated here by conducting some simulation scenarios in MATLAB. The employed parameters for the simulation study are given in Table 4. These parameters are selected according to Ref. [7] to check the effect of the proposed controller on the mentioned dermal vascular cancer tumour. However, it is proved later by simulation that according to Lyapunov theory, the proposed controller is stable and robust against the small changes in the values of this parameter for this specific type of cancer.

**TABLE 4:** Employed parameters of the simulation study

Concept	Numerical value	Parameters
NCs proliferation rate	0.0068 ${\text{day}}^{-1}$	$\alpha_{1}$
CCs proliferation rate	0.01 ${\text{day}}^{-1}$	$\alpha_{2}$
ECs proliferation rate	0.002 ${\text{day}}^{-1}$	$\alpha_{3}$
Chemical washout rate	$0.01813\;{\text{day}}^{-1}$	ξ
Anti‐angiogenic washout rate	$0.0136\;{\text{day}}^{-1}$	η
Saturation rate on NCs	1.10	$a_{1}$
Saturation rate on CCs	4.6205	$a_{2}$
Saturation rate on ECs	4.6666	$a_{3}$
NCs loss rate by CA	$1.2\times 10^{-7}\;{\text{day}}^{-1}$	$p_{10}$
Rate of ECs cooperation on CA for NCs	$2.4\times 10^{-4}\;{\text{day}}^{-1}$	$p_{11}$
Rate of AA cooperation on CA for NCs	$1.0\times 10^{-7}\;{\text{day}}^{-1}$	$p_{12}$
CCs loss rate by CA	$0.2051\;{\text{day}}^{-1}$	$p_{20}$
Rate of ECs cooperation on CA for CCs	$0.00431\;{\text{day}}^{-1}$	$p_{21}$
Rate of AA cooperation on CA for CCs	$19.4872\;{\text{day}}^{-1}$	$p_{22}$
ECs loss rate by AA	$1.7143\;{\text{day}}^{-1}$	$p_{3}$
Competitive rate of NCs	$0.00702\;{\text{day}}^{-1}$	$q_{1}$
Competitive rate of CCs	$0.00072\;{\text{day}}^{-1}$	$q_{2}$
CCs production rate due to ECs	$0.00371\;{\text{day}}^{-1}$	β
Coefficient of ECs for blood supply to the tumour	0.1615	γ

Abbreviations: AA, anti‐angiogenic agents; CA, chemotherapy agents; CC, cancer cell; EC, endothelial cell; NC, normal cell.

δ and ϕ are the related controlling signals representing the chemotherapy and anti‐angiogenic. In the case in which the chemotherapy is used as the sole therapy, ϕ (anti‐angiogenic) has a constant value, which is $2.4\times 10^{-4}\;{\text{day}}^{-1}$. Also, the initial conditions are:

$$x_{1}\left(0\right)=0.6,\;x_{2}\left(0\right)=0.6,\;x_{3}\left(0\right)=y\left(0\right)=w\left(0\right)=0$$

### 5.1 Controller results

**FIGURE 6:**
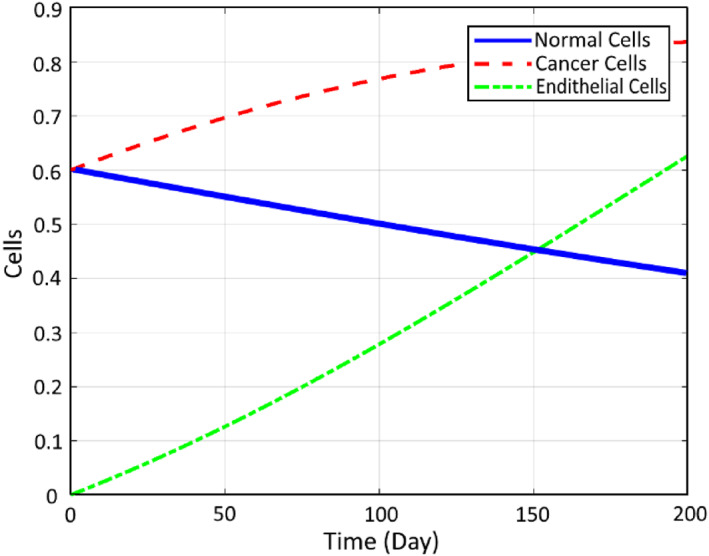
Open‐loop response of the cancer dynamics

First of all, in order to show the correctness of modelling, the pattern of the cancer cells growth is extracted for the open‐loop model of the cancer response while no controlling input is injected as shown in Figure 6.

It can be seen that the state related to the number of cancer cells is unstable and diverges to its saturation value with a settling time of about 500 days. This shows the correctness of the modelled dynamic of the cancer response and confirms the necessity of designing a properly closed‐loop controller.

**FIGURE 7:**
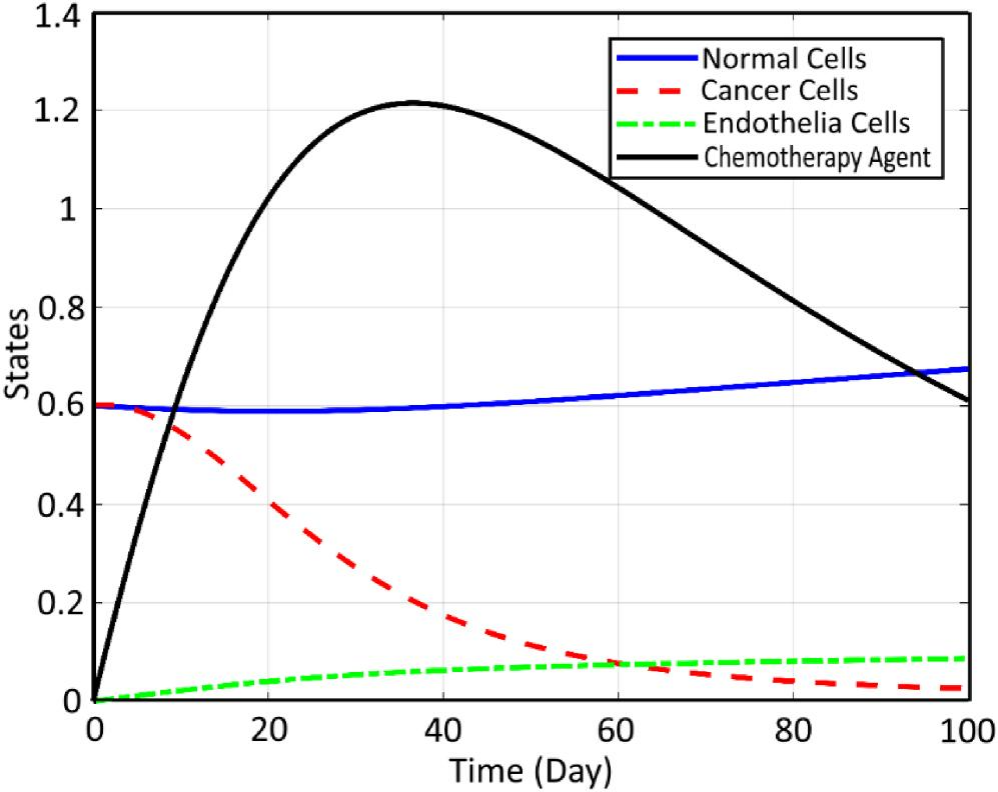
Chemotherapy agent

Considering CA therapy, pole placement is performed for the designed FL and the controlling coefficients are set as $k_{0}=6\;.\;k_{1}=5$, which results in the Eigen values of −2 and −3. The related profile of the states in the closed‐loop process of therapy is shown in Figure 7.

**FIGURE 8:**
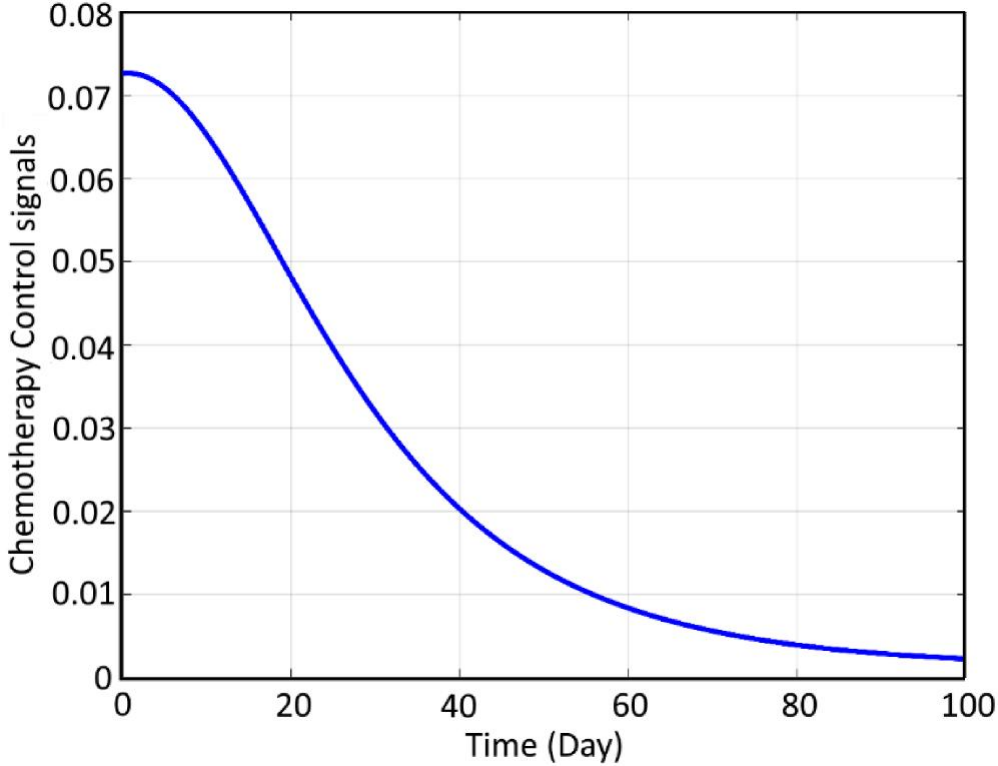
Chemotherapy control signal

It can be seen that using the proposed closed‐loop controller of FL, not only is the instability of the cancer cells blocked but also the normal cells are gradually increased and the cancerous ones are significantly decreased. The related input signal for the mentioned closed‐loop therapy based on chemotherapy can be observed in Figure 8:

It can be seen that as expected from a closed‐loop regulation process, the input signal is maximum at its initial stage where the cancer cells are maximum and gradually decreases as the cancer cells decrease. Also, the output trend of the system, which is the chemotherapy agent, can be observed as follows.

**FIGURE 9:**
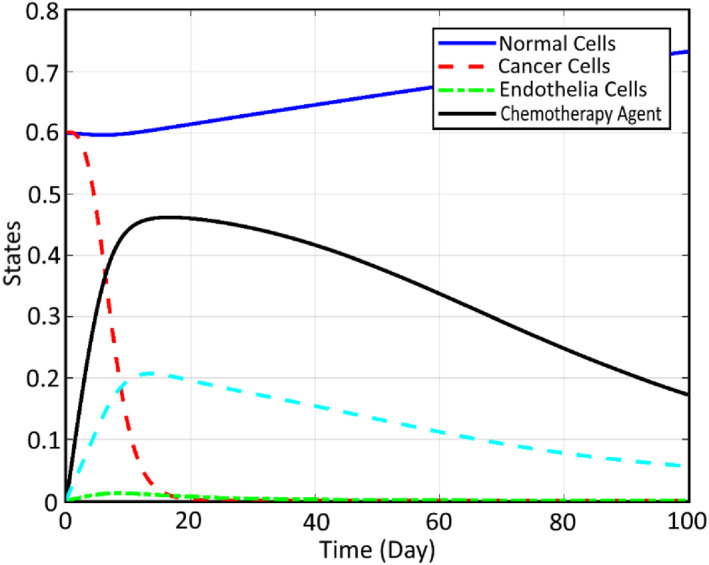
States with chemotherapy agents and anti‐angiogenic agents controller

It can be seen that the chemotherapy injection is increased rapidly during the first 50 days as a result of the FL controller in order to control the growth of the tumour and limit the metastases progress; however, the required injection is reduced gradually after the mentioned 50 days since the critical period of the cancer dynamics is passed during this range. Note that, the CCs are not completely destroyed and also the CA is too big and hurts the NCs too. Thus, it is proposed here to employ both the CA together with AA therapy to accelerate the treatment. Considering CA+AA therapy, the corresponding profiles of the states are extracted as shown in Figure 9.

Comparing the two mentioned methods, it can be concluded that the CCs are very small when the AA and CA are employed together and also, the EC is reduced satisfactorily. It is worth mentioning that when we use CA individually and the dose of the drug is low, then CCs get stronger and grow, but when AA is added to the CA, the corresponding CCs decrease because of the small value of the EC.

**FIGURE 10:**
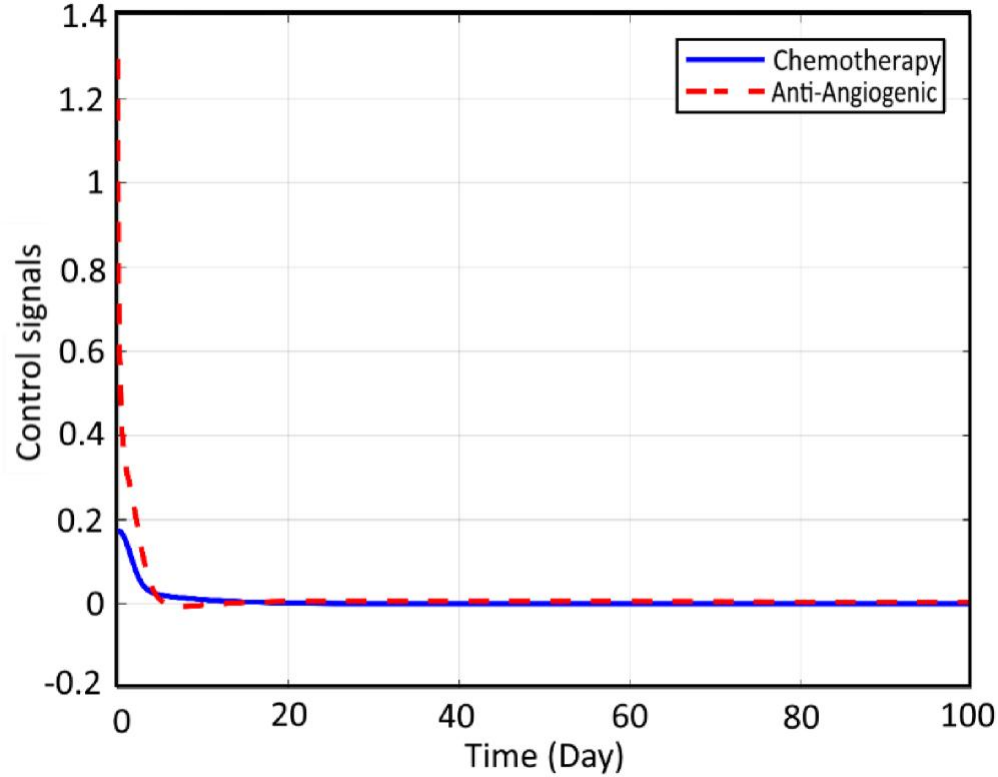
Input signals while using chemotherapy agents and anti‐angiogenic agents controller

**FIGURE 11:**
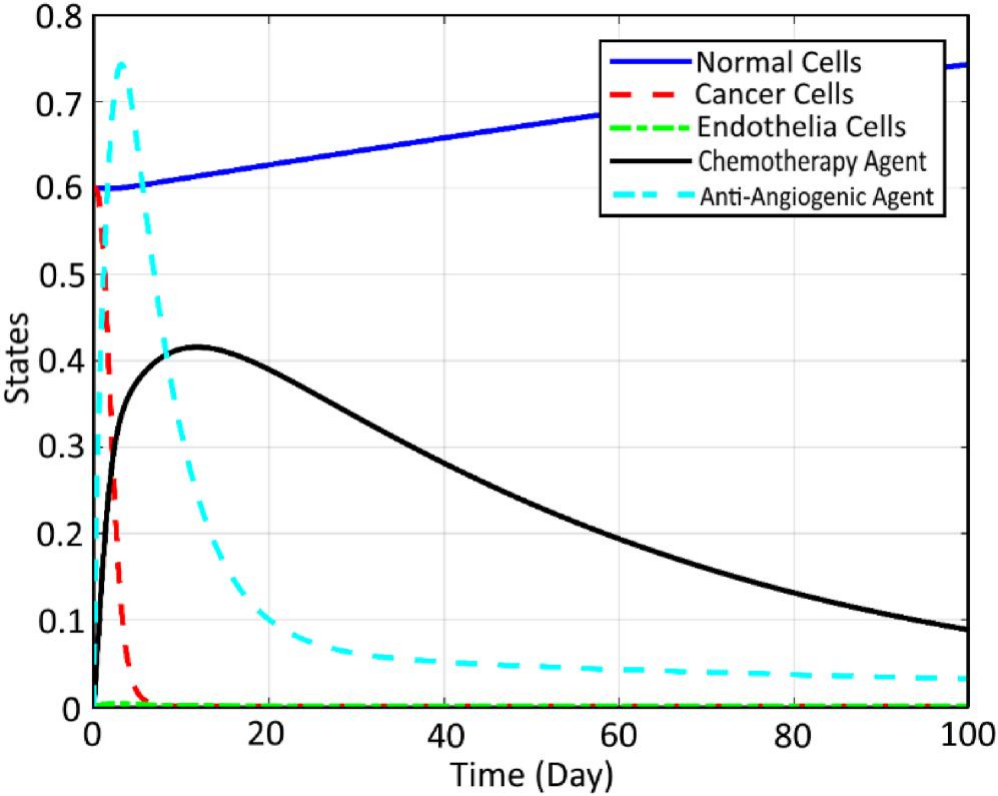
States using chemotherapy agents and anti‐angiogenic agents

Also, it can be concluded that the dose of the CA is reduced significantly here. However, it takes 100 days to destroy the CCs effectively. In Figure 10 corresponding controlling signals are used to realise the mentioned therapy and its related agent history are depicted:

Using the proposed modification by the aid of CA and AA and applying it to the system dynamics, the profiles of the states and inputs are improved, which can be seen in Figure 11.

It can be seen that the efficiency of therapy is increased significantly compared to Ref. [23] from the following three points of view:

1. The number of CCs are decreased to zero in about 30 days here, while in Ref. [23], the required time is more than 100 days, which shows the improvement of the settling time of the controlling process.
2. The amount of NCs is closer to 1 here compared to Ref. [10], which shows the faster treatment of the patient by using the proposed controller of this paper.
3. Less amount of chemotherapy injection is used here, which results in fewer side effects for the patient.

### 5.2 Optimising and increasing the robustness

**FIGURE 12:**
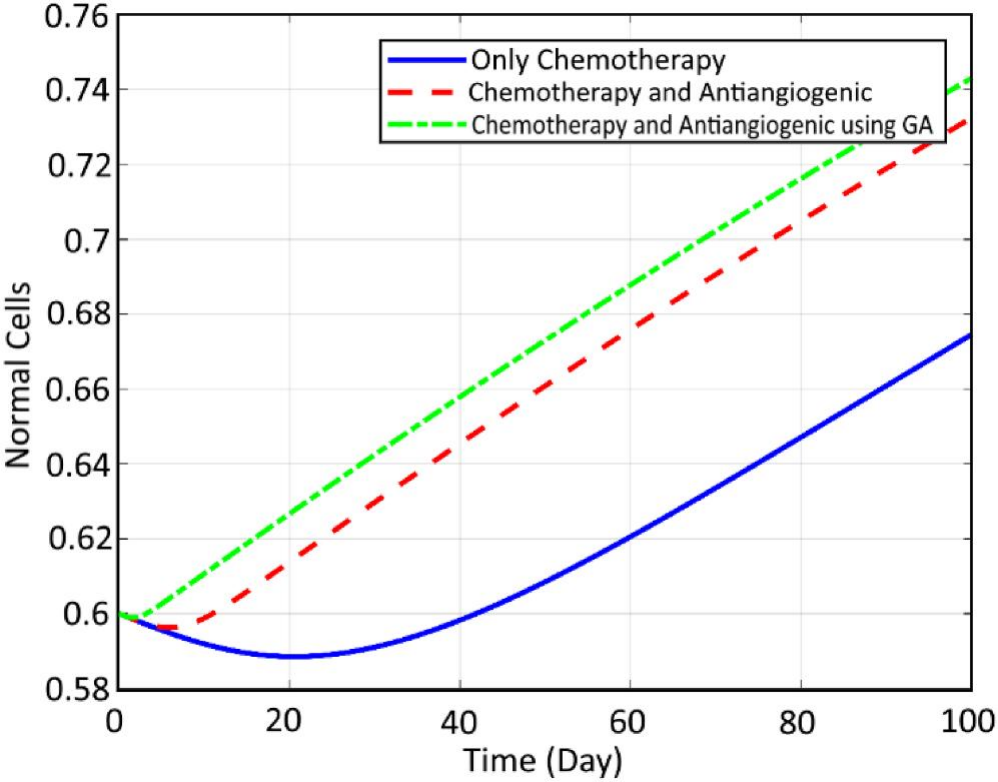
Comparison of the normal cells between the three mentioned methods

**FIGURE 13:**
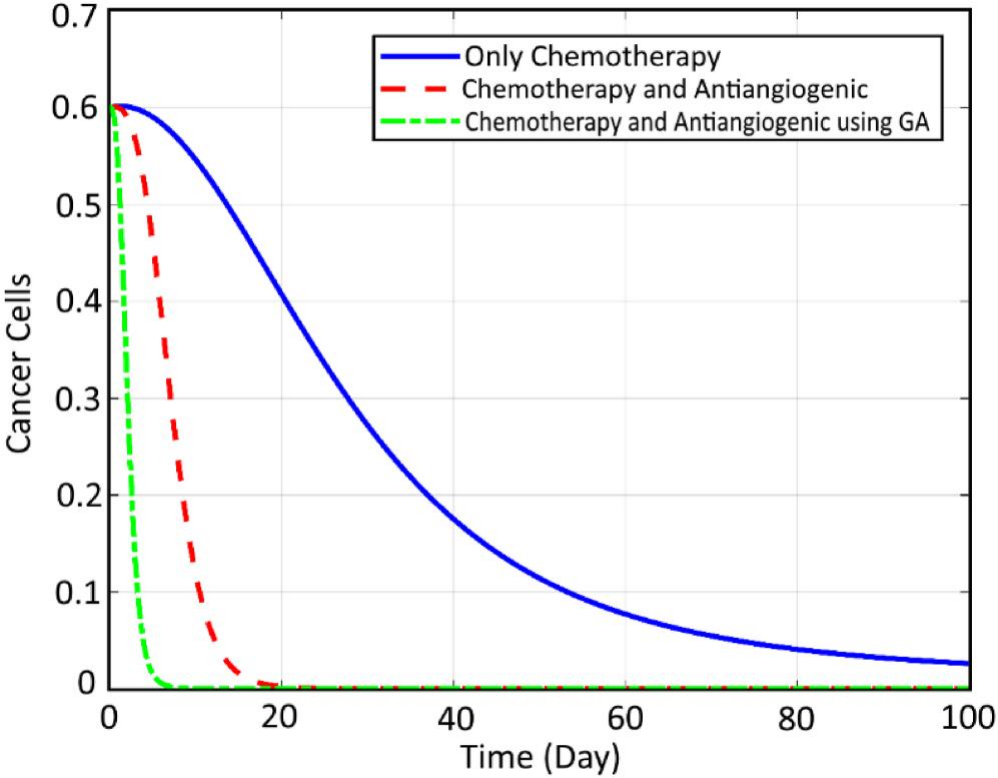
Comparison of the cancer cells between the three mentioned methods

**FIGURE 14:**
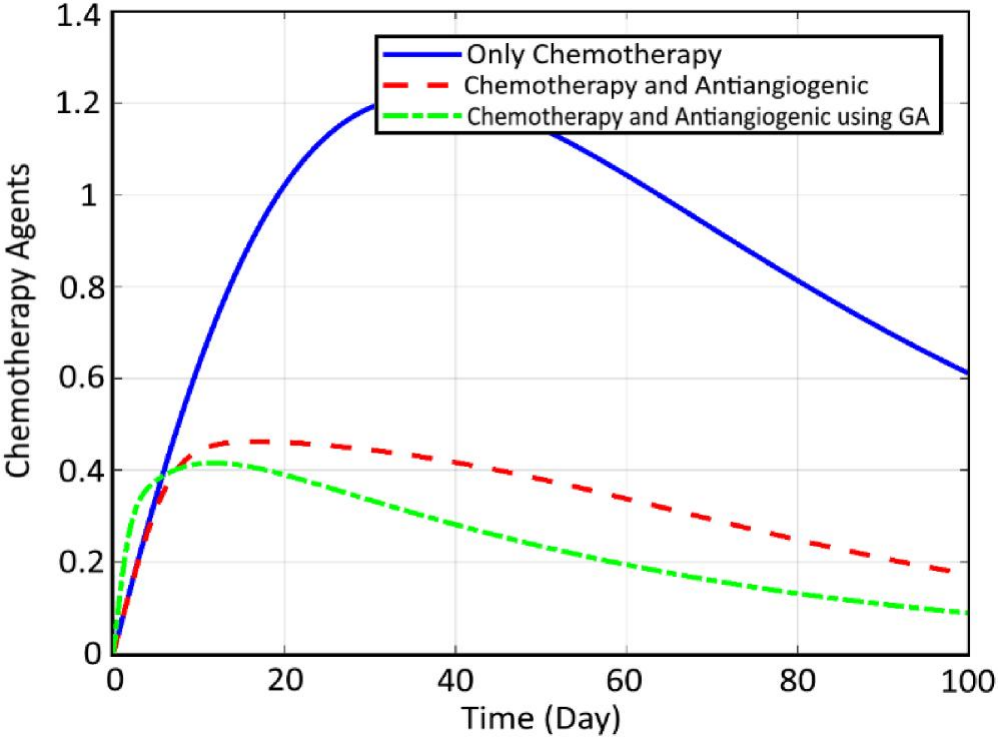
Comparison of the chemotherapy agent input between the three mentioned methods

**FIGURE 15:**
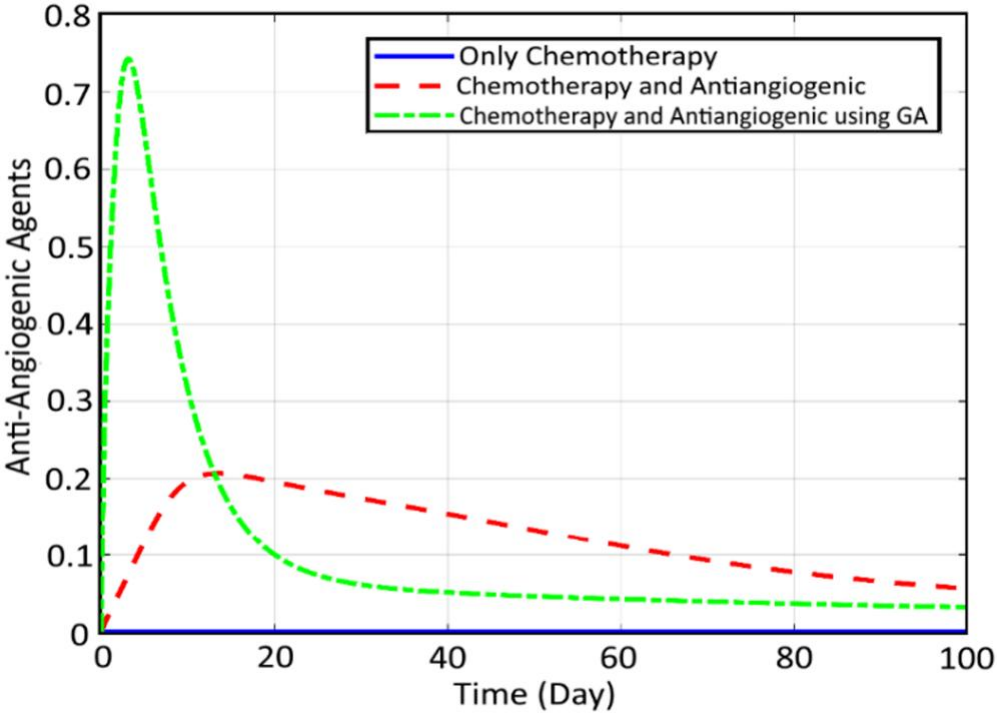
Comparison of the anti‐angiogenic agent input between the three mentioned methods

In order to check the efficiency of the proposed robust SMC controller and also optimisation tools for reduction of the cancer cells, some comparative simulations are conducted here. First, the comparison of the states and the inputs are performed here between the CA, CA+AA and the optimised CA+AA. Figure 12 compares the normal cells, while Figure 13 shows the comparison of cancer cells. It can be seen that for both profiles, the worst case is related to chemotherapy, while including the AA therapy has increased the therapy efficiency by about 96%, and the cancer cell, at last, converges to zero. Finally, the best response goes to the proposed closed loop FL controller optimised by GA, which has strengthened the treatment by about 95% and the cancer cell converges to zero before 30 days. Finally, Figures 14 and 15 illustrate this comparison for the chemotherapy agent input and the anti‐angiogenic agent input between the three mentioned algorithms, respectively.

As can be seen, the rate of the chemotherapy agent is decreased significantly (about 96%) as the result of using AA and the reduction is even increased by optimising it using GA. The required drug for the two other algorithms is roughly the same. However, the GA trend is faster, which results in a faster and more efficient decrease in cancer cells. The same trend can be observed for the input signal of anti‐angiogenic. Also, the rate number of the cancer cells is decreased obviously. It is worth noting that as a result of employing the proposed genetic optimisation, the reduction of the cancer cells to zero is realised within a limited time of about 30 days, while two other methods meet this goal within an infinite time duration. Moreover, the corresponding rate of the normal cells is increased up to about 1 for the optimised controller.

**FIGURE 16:**
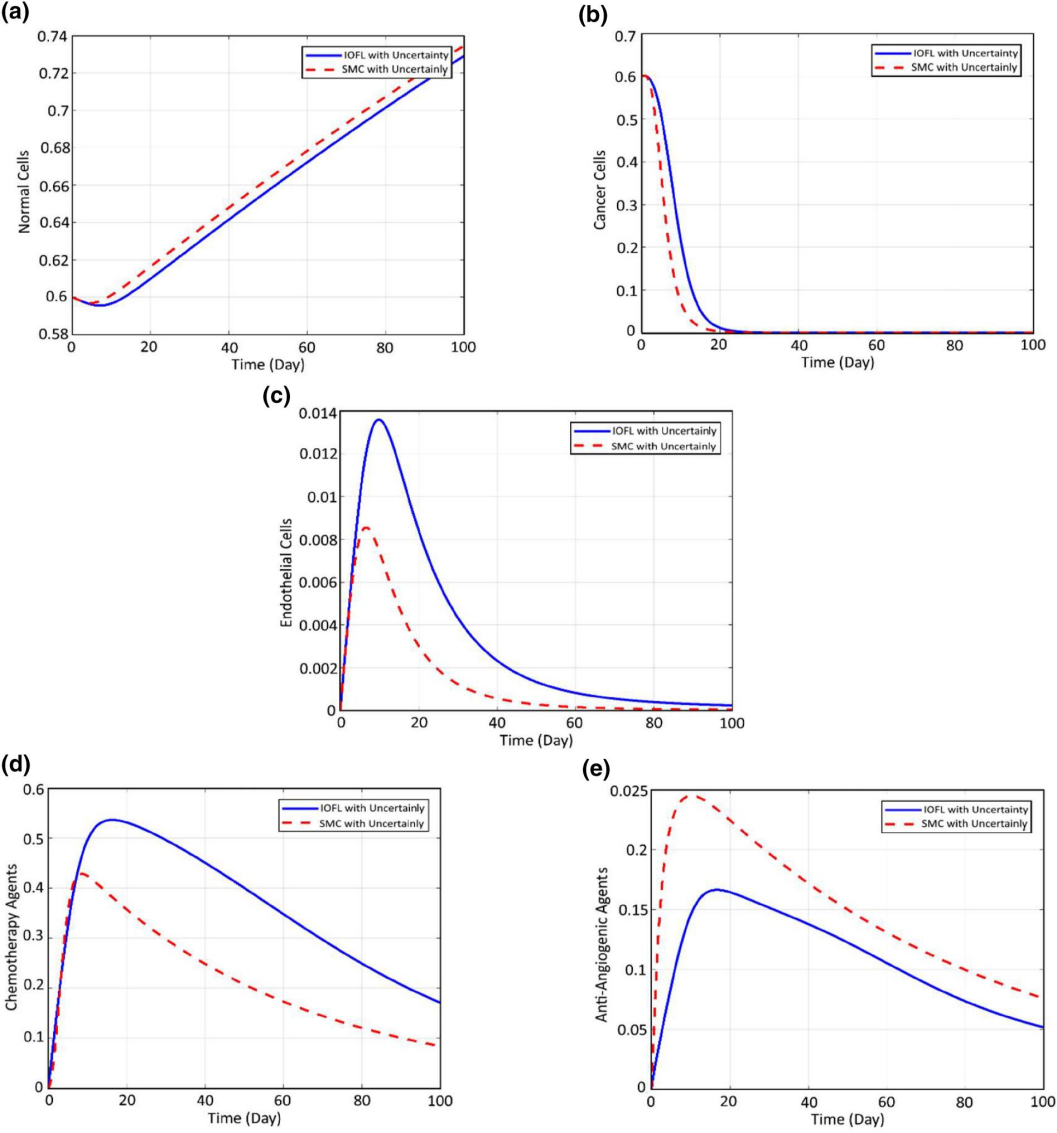
Comparison of the performance of the cancer dynamic, which is controlled using feedback linearisation and sliding mode in the presence of 20% parametric uncertainty

The comparison of the resultant values for the considered cost function between the mentioned algorithms can be seen in the following table. As can be seen from this table, first of all, employing the proposed anti‐angiogenic input has decreased the rate of injection 95%, which consequently decreases the inevitable side effects of the chemotherapy. Using anti‐angiogenic injection, the rate of chemotherapy injection is a little lower than the case the genetic algorithm is employed. However, optimising the controlling input with the aid of this optimisation tool has caused the death of the majority of the cancer cells during 1 month. Indeed, the implementation of genetic optimisation tool results in more usage of anti‐angiogenic input and increasing the efficiency of chemotherapy. In the second case, the minimum amount of chemotherapy injection is used while the cancer cells are destroyed. Finally, in the last case, the injection rate is a little more, but since a proper amount of anti‐angiogenic is engaged, the efficiency of the treatment increases and causes that the cancer cells could be destroyed during a limited time. Comparison of the performance of the cancer dynamicbetween feedback linearisation and sliding mode in the presence of 20% parametric uncertainty can be seen in Figure 16.

In order to check the efficiency of the proposed robust controller to treat cancer in the presence of parametric uncertainties related to the impact rate of the chemotherapy and anti‐angiogenic drug on the cells, the dynamic response of the cancer cells and the corresponding required controlling input of chemotherapy drug and anti‐angiogenic is compared here between the two controllers of feedback linearisation and sliding mode for which in both cases, the gains are tuned, using the optimisation method of genetic.

Considering the presented parameters in Table 5, it is supposed that the parameters $p_{21},p_{3},p_{20},p_{22}$ have uncertainty about 20%.

**TABLE 5:** Parameters with uncertainty

$p_{20}$	The rate of cancer cells destroyed with the aid of chemotherapy drug
$p_{21}$	The rate of cooperation of endothelial cells with chemotherapy drug for the cancer cells
$p_{22}$	The rate of cooperation of anti‐angiogenic with chemotherapy drug for the cancer cells
$p_{3}$	The rate of endothelial vessels destroyed with the aid of anti‐angiogenic drug

It can be seen that the settling time of the system in which the cancer cells are controlled using sliding mode is less than the one which is equipped with simple feedback linearisation, which means that the cancer cells will be destroyed sooner. Moreover, the required chemotherapy drug and anti‐angiogenic are decreased by about 18% using the robust controller of sliding mode, which consequently results in lower drug side effects in the patient's body.

**FIGURE 17:**
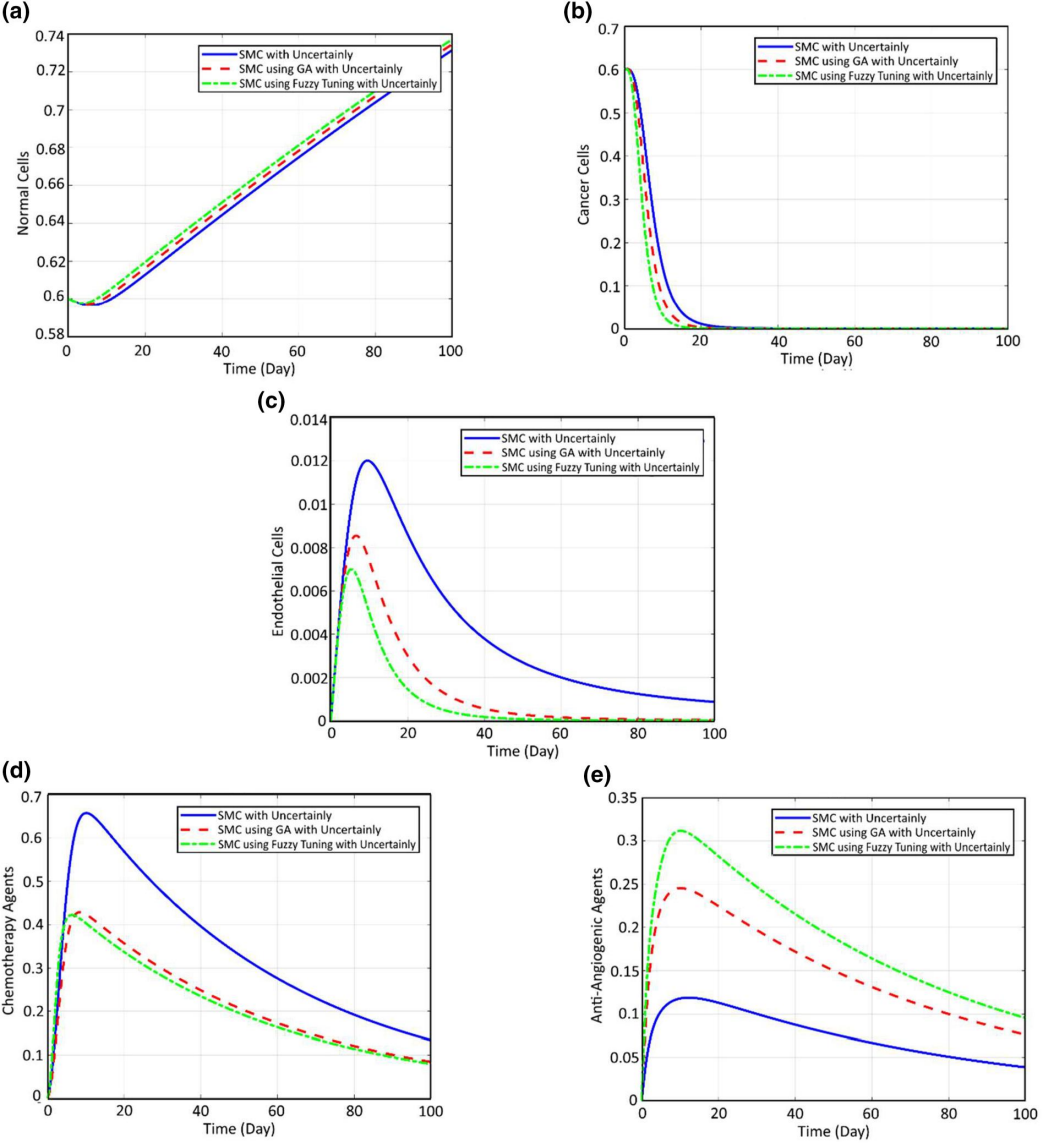
Comparing the cancer response in the presence of uncertainties between simple sliding mode, sliding mode with genetic tuner, and the sliding mode with fuzzy tuner

Finally, in order to verify the efficiency of the proposed tuning of the controller gains and study the advantages and disadvantages of the fuzzy and genetic tuners, the cancer response in the presence of the mentioned parametric uncertainties is compared between the simple sliding mode, a sliding mode in which the controllers are tuned using genetic algorithm and the one which is tuned using the fuzzy algorithm (Figure 17). It was shown previously that the sliding mode has a more efficient impact of the cancer treatment compared to feedback linearisation. It can be seen now, according to Figure 17, tuning the sliding mode gains using genetic or fuzzy algorithms can even decrease the required chemotherapy injection up to 33% with lower side effects and higher treatment response.

Comparing the fuzzy tuner and the genetic one, it can be concluded that since in the case that the gains are tuned using the fuzzy algorithm, the anti‐angiogenic drug is more, the cancer cells can be destroyed sooner with the aid of the same dose of chemotropic drug and thus the patient can be cured sooner. The two controllers of feedback linearisation and sliding mode were designed and implemented on the cancer tumour dynamics using anti‐angiogenic, and the performance of the patient cure was compared in the presence of parametric uncertainties. It was seen that the sliding mode provides a stronger treatment as it was expected about 41.3%.

Comparing the results of the proposed treatment with the one which is designed in Ref. [23], it can be seen that here the cancer cells converge to zero faster than Ref. [23] and its settling time is about 50%. Also, the required injected chemotherapy is three times less than the proposed treatment of Ref. [23].

**FIGURE 18:**
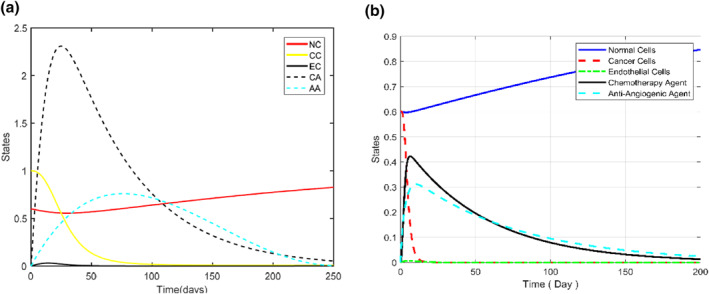
(a) Diagram of states shown in Ref. [23]. (b) Diagram of states based on the proposed sliding mode control and fuzzy tuning

In the following chart, the main critical characteristics of Figure 18 are compared between the proposed controller and the treatment of Ref. [23]. It can be seen accordingly that the proposed controller of this paper can significantly improve the treatment process of Ref. [23].

Tuning the controlling gains can increase the efficiency of the treatment by about 33%. Also, the gain tuners of fuzzy and genetic algorithm were compared and it was seen that using anti‐angiogenic, the fuzzy tuner can cure the patient faster and with a lower amount of side effects by about 3.5%. The comparison of the efficiency related to all the mentioned methods, on the therapy objective function, can be seen in Table 6. Here the best performance is related to the SMC controller with the fuzzy tuner.

**TABLE 6:** Comparison parameters of Figure 18

Article	Maximum chemotherapy agent	Maximum anti‐angiogenic agent	Time of cancer cells converges to zero
This article	0.4	0.3	Less than 25 days
[23]	2.2	0.7	More than 50 days
[31]	1.7	7	Less than 25 days

To sum up, in the presence of parametric uncertainties, when FL is optimised, the improvement is not significant. But it can be seen that for the studied case, the optimised SMC can improve the treatment up to 40%, which is a valuable gain. Please note that in chemotherapy any little improvement and acceleration in therapy is highly significant since the faster the recovery, the lower the treatment side effect. Thus as a result of the proposed optimisation in control, the recovery can take pace faster and the chemotherapy can be blocked sooner and the patient can get rid of its related high side effects.

In Table 7, all of the designed controllers, including feedback linearisation without GA, feedback linearisation with GA, SMC with GA, and SMC with fuzzy tuning, are compared in terms of chemotherapy drug injection, anti‐angiogenic injection, and also the percentage of patient improvement with respect to the previous methods.

**TABLE 7:** Comparison of the objective function for different therapies

Row	Method of control	Dates to kill cancer cells	Average of chemotherapy injection per day	Total amount of chemotherapy in 250 days	Average of anti‐angiogenic injection per day	Total amount of anti‐angiogenic in 250 days	Improvement percent respect to the previous method
1	Feedback linearisation without GA	More than 300 days (never goes zero)	0.2293	4586	0.0793	1587	N/A
2	Feedback linearisation with GA	More than 24 days (converges to zero in infinite time)	0.2192	4384	0.0699	1399	%4.4
3	SMC with GA	Less than 20 days (converges to zero in finite time)	0.1286	2572	0.0973	1945	%41.33
4	SMC with fuzzy tuning	Less than 15 days (converges to zero in finite time)	0.1241	2481	0.1219	2438	%3.53

Abbreviation: GA, genetic algorithm.

## 6 CONCLUSION

In this paper, a model of the cancer metastases was presented and the dynamics of the cancer cells growth were estimated analytically based on Ref. [7]. Afterwards, two non‐linear closed‐loop controllers were designed and implemented on the modelled biological system based on FL and SMC. It was observed that the cancer cells could be destroyed using CA and CA+AA effectively. Also, it was shown that using the robust method of SMC, the parametric uncertainties of the patient tumour model could be effectively neutralised for more than 40% parametric uncertainty. Afterwards, the mentioned controlling algorithm was optimised using GA and fuzzy. To do so, the related coefficients of the designed controllers were tuned optimally. It was observed that the optimum controllers decreased the side effect of treatment by about 41.33%. Also comparing the fuzzy tuner and genetic algorithm, it showed 3.53%. It was shown that, although the improved method of CA+AA could decrease the number of cancer cells, it could not vanish them perfectly to zero. It was observed that in using the methods of chemotherapy and anti‐angiogenic, the minimum amount of chemotherapy injection was used while the cancer cells were destroyed. However, in the proposed optimal control in which the combination of chemotherapy and anti‐angiogenic is employed, the injection rate is a little more, but since a proper amount of anti‐angiogenic is engaged, the efficiency of the treatment increased and caused that the cancer cells could be destroyed during a limited time. In order to show the efficiency of the proposed controlling treatment, the cancer cells and required injection were compared with previous studies. It was seen that the settling time based on the proposed treatment was about half of the mentioned references and the injection was about one‐third of these references. Therefore, using the proposed optimisation tool of GA, not only can the chemotherapy agent dosage be reduced by about 96% but also the cancer cells can be destroyed completely during a limited time of 30 days. Thus, it can be concluded that the proposed optimised controller can successfully control the cancer progression within a limited time.

## AUTHOR CONTRIBUTIONS

**Ehsan Sadeghi Ghasemabad:** Conceptualization, Methodology, Software, Writing ‐ Review and Editing. **Iman Zamani:** Conceptualization, Methodology. **Hami Tourajizadeh:** Scientific Consulting, Writing‐original draft, Writing ‐ Review and Editing. **Mahdi Mirhadi:** Methodology, Software, Writing ‐ Review and Editing. **Zahra Goorkani Zarandi:** Methodology, Software, Writing ‐ Review and Editing.

## CONFLICT OF INTEREST

There is no conflict of interest to declare.

## Data Availability

The data that support the findings of this study are available from the corresponding author upon reasonable request.
